# Expression and role of the immune checkpoint regulator PD-L1 in the tumor-stroma interplay of pancreatic ductal adenocarcinoma

**DOI:** 10.3389/fimmu.2023.1157397

**Published:** 2023-06-28

**Authors:** Tina Daunke, Silje Beckinger, Sascha Rahn, Sandra Krüger, Steffen Heckl, Heiner Schäfer, Daniela Wesch, Christian Pilarsky, Markus Eckstein, Arndt Hartmann, Christoph Röcken, Anna Maxi Wandmacher, Susanne Sebens

**Affiliations:** ^1^Institute for Experimental Cancer Research, Kiel University and University Hospital Schleswig-Holstein, Kiel, Germany; ^2^Biochemical Institute, Kiel University, Kiel, Germany; ^3^Institute of Pathology, University Medical Center Schleswig-Holstein, Kiel, Germany; ^4^Department of Internal Medicine II, University Medical Center Schleswig-Holstein, Kiel, Germany; ^5^Institute of Immunology, Kiel University and University Medical Center Schleswig-Holstein (UKSH), Kiel, Germany; ^6^Translational Research Center, University Hospital Erlangen, Erlangen, Germany; ^7^Institute of Pathology, University Hospital Erlangen, Friedrich-Alexander-Universität Erlangen-Nürnberg (FAU), Erlangen, Germany

**Keywords:** pancreatic cancer, immune checkpoint inhibitor, immune evasion, tumor microenvironment, macrophages, 3D co-culture

## Abstract

**Introduction:**

Immune checkpoint inhibitors (ICI), e.g., targeting programmed cell death protein 1-ligand 1 (PD-L1) or its receptor PD-1, have markedly improved the therapy of many cancers but so far failed in pancreatic ductal adenocarcinoma (PDAC). Macrophages represent one of the most abundant immune cell populations within the tumor microenvironment (TME) of PDAC being able to either support or restrain tumor progression depending on their phenotype. To better understand treatment failure of PD-L1/PD-1 inhibitors in PDAC, this study examined PD-L1 expression in the context of a dynamic TME in PDAC with a particular focus on the impact of macrophages.

**Methods:**

Formalin-fixed and paraffin embedded tissue samples of primary PDAC tissues and corresponding liver metastases were used for immunohistochemical analyses. Serial sections were stained with antibodies detecting Pan-Cytokeratin, CD68, CD163, CD8, and PD-L1.To investigate whether the PD-1/PD-L1 axis and macrophages contribute to immune escape of PDAC cells, a stroma enriched 3D spheroid coculture model was established in vitro, using different PDAC cell lines and macrophages subtypes as well as CD8+ T cells. Functional and flow cytometry analyses were conducted to characterize cell populations.

**Results:**

Immunohistochemical analyses revealed that PD-L1 is mainly expressed by stroma cells, including macrophages and not PDAC cells in primary PDAC tissues and corresponding liver metastases. Notably, high local abundance of macrophages and strong PD-L1 staining were commonly found at invasion fronts of tumoral lesions between CD8+ T cells and tumor cells. In order to investigate whether PD-L1 expressing macrophages impact the response of PDAC cells to treatment with PD-L1/PD-1 inhibitors, we developed a spheroid model comprising two different PDAC cell lines and different ratios of in vitro differentiated primary M1- or M2-like polarized macrophages. In line with our in situ findings, high PD-L1 expression was observed in macrophages rather than PDAC cells, which was further increased by the presence of PDAC cells. The effector phenotype of co-cultured CD8+ T cells exemplified by expression of activation markers and release of effector molecules was rather enhanced by PDAC macrophage spheroids, particularly with M1-like macrophages compared to mono-culture spheroids. However, this was not associated with enhanced PDAC cell death. ICI treatment with either Durvalumab or Pembrolizumab alone or in combination with Gemcitabine hardly affected the effector phenotype of CD8+ T cells along with PDAC cell death. Thus, despite strong PD-L1 expression in macrophages, ICI treatment did not result in an enhanced activation and cytotoxic phenotype of CD8+ T cells.

**Conclusion:**

Overall, our study revealed novel insights into the interplay of PDAC cells and macrophages in the presence of ICI.

## 1 Introduction

In western countries, pancreatic ductal adenocarcinoma (PDAC) is the 4^th^ leading cause of cancer-related deaths, with a low 5-year survival rate of 10% (1). PDAC is characterized by a pronounced immunosuppressive tumor microenvironment (TME) which plays a crucial role in tumor development and progression in the primary as well as in the secondary context (2). One of the most abundant stroma cell populations are tumor-associated macrophages (TAM) (3, 4) which can exhibit different phenotypes. Following a simplified model, an M1-like phenotype is associated with pro-inflammatory and anti-tumorigenic properties, while M2-like polarized macrophages exert anti-inflammatory and pro-tumorigenic effects (3). Macrophages are early recruited into precursor/tumoral lesions and, therefore, accumulate as one of the first immune cell populations (5). TAM’s frequency and effector phenotype are highly variable and change during PDAC progression. However, most TAM exhibit M2-like characteristics (6, 7), a feature associated with poor prognosis and metastasis (8, 9). Of note, PDAC-derived TAM exhibit properties of both M2- and M1-like polarization and have been shown to promote epithelial-mesenchymal transition (EMT), a process that fosters invasion of PDAC and premalignant pancreatic ductal epithelial cells (PDEC) (10). As EMT is also associated with the acquisition of a drug resistant phenotype and immune evasive properties, TAM are potent drivers of drug resistance as well as immune escape and suppression at all stages of tumor development (11).

One mechanism of immunosuppression and immune evasion of cancers is the highly aberrant expression of immune checkpoint molecules such as programmed cell death protein 1-ligand 1 (PD-L1) or its receptor programmed cell death protein 1 (PD-1). Accordingly, engagement of PD-1 on T cells by its ligands PD-L1 or PD-L2 on antigen presenting cells or tumor cells leads to T cell exhaustion (12–14). Interfering with this inhibitory axis by treatment with immune checkpoint inhibitors (ICI) such as Pembrolizumab or Durvalumab revolutionized the therapeutic regimens for multiple cancer entities (15–17), but so far failed in PDAC (18, 19), although considerable PD-L1 expression levels in PDAC cell lines has been reported in PDAC (20–22). In this context, Rahn et al. demonstrated that in PDAC tissues staged T3N1M0, representing the most common resected PDAC stage, PD-L1 is expressed only in one third of the cases and stromal cells such as TAM predominantly exhibit PD-L1 expression (23, 24). Still, it is poorly understood whether PD-L1 expressing TAM with different polarization phenotypes contribute to immune evasion of PDAC cells, particularly when the latter themselves do not or only hardly express PD-L1.

In order to obtain further insight into PD-L1-mediated immune evasion during PDAC progression and the reasons for anti-PD-L1 treatment failure in this tumor entity, this study examined PD-L1 expression and its spatial distribution in primary PDAC tissues and corresponding liver metastases. To mimic this dynamic TME comprising different amounts and phenotypes of macrophages, one of the main immune cell populations impacting anti-tumor immunity during PDAC progression, a stroma enriched spheroid model with two different PDAC cell lines and different ratios of M1- or M2-like macrophages was used. Using this model the present study particularly focused on how the interplay of macrophages and PDAC cells affect the activation and effector phenotype of CD8+ T cells as well as PD-L1 expression of macrophages and PDAC cells. Finally, it was elucidated how these distinct stromal conditions impact treatment with ICI in monotherapy as well in combination with Gemcitabine. Combining a comprehensive *in situ* analysis of primary and metastatic PDAC tissues with 3D stroma enriched PDAC cell cultures, our study provides novel insights in the role of macrophages and the PD-L1/PD-1 axis in immune evasion of PDAC.

## 2 Material and methods

### 2.1 PDAC patient information and histological analysis

Immunohistochemical (IHC) stainings of whole mount serial sections from primary tumor and corresponding liver metastases of four PDAC patients were performed. All tissue samples were surgical resects. Three out of four cases were untreated at time point of surgical resection and one patient had obtained neoadjuvant therapy. The research was approved by the ethics committee of Kiel University (reference number: A110/99). Written consent was obtained from all patients. Patient characteristics are listed in **Table 1**. For IHC stainings following antibodies were used: PanCK (dilution 1:200, clone: AE1/AE3, NeoMarkers *via* Thermo Fisher Scientific, Waltham, USA), PD-L1 (dilution 1:100, clone: E1L3N, Cell signaling Technologies, Danvers, USA), CD68 (dilution 1:100, clone: 514H12, Leica Microsystems GmbH, Wetzlar, Germany), CD163 (dilution 1:100, clone: 10D6, Leica Microsystems GmbH) and CD8 (dilution 1:100, clone: C8144B, Leica Microsystems GmbH). Immunohistochemical staining was performed with the autostainer Bond™ RX System.

**Table 1:** Clinic-pathological characteristics of PDAC patients in analyzed cohort of this study.

Parameter	Number of cases
Patients	4
Median age (range)	71 (68-74)
Sex (male/female)	3/1
Tumor stage T1/T2/T3/T4	0/2/2/0
Nodal stage N0/N1	1/3
Metastasis stage M0/M1	0/4
Tumor grade 1 (well differentiated)	1
Tumor grade 2 (moderately differentiated)	0
Tumor grade 3 (poorly differentiated)	3

Furthermore, IHC double stainings of liver metastases were performed. The first step involved the staining of PD-L1 (dilution 1:100, clone: E1L3N, Cell Signaling Technologies). Antigen retrieval was achieved with ER2 (EDTA-buffer Bond pH 9.0; 20 minutes). The antigen retrieval step was modified for the PD-L1 staining of those slides, which were to be combined with an alpha-smooth muscle actin (αSMA) staining in the second step: In those cases, we decided to enhance PD-L1 visualization in relation to the naturally intense αSMA signal by prolonging ER2 antigen retrieval to 30 minutes. The immunoreaction was visualized with the Bond™ Polymer Refine Detection Kit (DS 9800, brown labeling, Novocastra; Leica Microsystems GmbH) resulting in a brown color. The second step involved the staining of either αSMA (dilution 1:400, clone: 1A4, NeoMarkers *via* Thermo Fisher Scientific), or CD68 (dilution 1:100; clone: 514H12, Leica Microsystems GmbH), or PanCK (dilution 1:200, clone: AE1/AE3, NeoMarkers *via* Thermo Fisher Scientific). Antigen retrieval was carried out with ER1 (citrate buffer Bond pH 6.0; 20 minutes for αSMA), or ER2 (EDTA-buffer Bond pH 9.0; 20 minutes for CD68). The immunoreaction was visualized with the BONDT^M^ Polymer Refine Red Detection Kit (DS9390, red labeling, Leica Microsystems GmbH) resulting in a red color.

Stained tissue sections were scanned using a Hamamatsu NanoZoomer 2.0 RS scanner (Hamamatsu Photonics Deutschland GmbH, Herrsching am Ammersee, Germany) at 400 times magnification and viewed with NDP.view2 software (Hamamatsu photonics). Analyses of the single IHC stainings were performed in two steps. To analyze the cellular and histoanatomical distribution of different stained markers, an overall evaluation of the tissue sections was first performed: the entire tissue section was screened at low magnification and the predominant distribution of immunopositive cells (tumor center and/or invasion front of primary tumor or liver metastases) was documented. In order to investigate more precisely the distribution and frequency of staining, ten representative fields of view (FoV) at the invasion front and tumor center (defined as at least one FoV away from invasion front at 200-fold magnification) of the primary tumor or metastasis were investigated. All tissue sections were studied with regard to the predominant areas of stained cells at 200-fold magnification (FoV: 0.48x0.85 mm), except those stained for PD-L1 which were analyzed at 400-fold magnification (FoV: 0.42x0.24 mm). Every tissue section was assessed by their predominant localization of immunopositive cells (at the invasion front, tumor/metastasis center or evenly distributed between invasion front and tumor center). For staining evaluation, a scoring system was established which rates the percentage of immunopositive cells in the FoV. For scoring of highly abundant immunopositive cells (CD163+ as well as CD68+ macrophages), cell frequency was scored as 0%, <10%, 11-50% and >50% positively stained cells. As these categories were not suitable for less abundant immunopositive stained cells like CD8+ T cells and PD-L1 staining, PD-L1 and CD8 staining were graded into 0% (negative), <1% and > 1% positively cells stained. Furthermore, the PD-L1 staining intensity was graded as none, low and high. For each tissue section, areas with the highest frequency of immunopositive cells were documented to evaluate the main localization of cells stained for the respective markers within the tumor tissue. All stainings were evaluated independently by two examiners (SB and TD).

### 2.2 Generation of macrophages

Lymphocytes and monocytes were isolated from leukoreduction system chambers of healthy blood donors provided by the Institute of Transfusion Medicine in Kiel. Written informed consent from all donors was obtained. Isolation of peripheral blood mononuclear cells (PBMC) was done by density gradient centrifugation followed by counterflow centrifugation to separate lymphocytes from monocytes according to established protocols (10, 25). All lymphocyte fractions above 85% purity were frozen and only monocytes from fractions with a monocyte purity higher than 85% were polarized into M1- or M2-like macrophages as previously described (26). For this purpose, monocyte fractions were centrifuged and resuspended in M2 medium (RPMI-1640 medium, 1% FCS, 1% L-glutamine, 1% penicillin/streptomycin) and the cell number was determined. Then, 2x10^6^ monocytes per well were seeded into 12-well plates, stimulated either with 2.4 ng/ml GM-CSF (Biolegend, San Diego, USA) for M1-like macrophage polarization or 50 ng/ml M-CSF (Biolegend) for M2-like macrophage polarization and cultured for 6 to 7 days at 37°C, 5% CO_2_ and 86% relative humidity. Culture medium for induction of M1-like polarization was supplemented with 5% fetal calf serum (FCS), while culture medium for induction of M2-like polarization was supplemented with only 1% FCS. Polarization into M1- and M2-like macrophages was verified according to established protocols with confirmed polarization markers (10, 27, 28).

### 2.3 Isolation of human CD8+ T cells

Frozen human lymphocytes derived from counterflow centrifugation were thawed in T cell medium (TCM) (RPMI-1640 medium, 10% FCS, 1% L-glutamine, 1% penicillin/streptomycin, 1% Sodium pyruvate and 2% HEPES). After centrifugation, cell count was determined. CD8+ T cells were isolated using the magnetic activated cell sorting (MACS) isolation kit (Miltenyi, Bergisch Gladbach, Germany) following modified manufacturer’s instructions with reduced antibody and bead concentrations (50%) and extended incubation times (1.5-fold). After negative selection, CD8+ T cells were counted and analyzed for their purity *via* flow cytometry (MACSQuant X, Miltenyi) using immunofluorescence staining with the following antibodies: αβ-TCR-FITC (clone: IP26, Biolegend), CD4-APC (clone: RPA-T4, Biolegend) and CD8-PE (clone: RPA-T8, Biolegend). Only T cell populations with a purity higher than 90% were used for further experiments.

### 2.4 Activation of human CD8+ T cells

Prior to co-culture experiments, activation of isolated naïve CD8+ T cells was performed. For this purpose, a 24-well plate was coated by incubation for 2 h at 37°C with 200 µl of 1.5 µg/ml anti-CD3 antibody (clone: OKT3, Biolegend) in PBS. Before T cells were seeded, wells were washed 2-times with PBS. Then, 1.3 – 1.8x10^6^ naïve CD8+ T cells were seeded in 1 ml TCM supplemented with 1.5 µg/ml anti-CD28 antibody (clone: CD28.2, Biolegend) and 60 ng/ml IL-2 (Biolegend). CD8+ T cells were cultured for 3 days at 37°C, 5% CO_2_ and 86% humidity.

### 2.5 Cultivation of human PDAC cell lines

Panc89 cells, derived from a lymph node metastasis of a 64-year-old caucasian male (29–31), were used as a PDAC cell line with low PD-L1 expression. Panc89 cells are described to be moderately differentiated and exhibit a mutation in *p53* (T220C) and depletion of *p16*, while the *k-ras* and *SMAD4* genes show a wildtype status (29, 30). PancTu1 cells, derived from the primary tumor of a caucasian female (29, 30), were used as a PDAC cell line with moderate PD-L1 expression. PancTu1 cells are described to be poorly differentiated and exhibit mutations in *k-ras* (G12V) and *p53* (C176S) and a depletion of *p16*. *SMAD4* have a wildtype status (29, 30). Both cell lines were cultivated in 75 cm^2^ cell culture flasks with 10 ml medium (RPMI-1640 medium, 10% FCS, 1% L-glutamine, 1% Sodium pyruvate) at 37°C, 5% CO_2_ and 86% humidity.

### 2.6 CellTracker labelling

In order to track and discriminate PDAC cells from macrophages during 3D co-culture, cell populations were labeled with different cell-trackers before seeding in mono- or co-culture spheroids. Tumor cells were labeled with CellTracker green (Invitrogen, Waltham, USA) and macrophages with CellTrace violet (Invitrogen) following the manufacturer’s instructions. Visualization of the cells within spheroids was performed with a Lionheart FX microscope (Agilent BioTek, Santa Clara, USA).

### 2.7 Mono- and co-culture spheroids

For spheroid formation, a total cell number of 2x10^4^ cells per well was seeded in 96-well ultra-low attachment plates (Biofloat 96 well-plate, faCellitate, Mannheim, Germany). For mono-culture spheroids, 2x10^4^ PDAC cells were seeded. For co-culture spheroids with a 1:1 PDAC cell/macrophage ratio, 1x10^4^ PDAC cells and 1x10^4^ polarized macrophages were seeded. in case of a 3:1 ratio, 1.5x10^4^ PDAC cells and 0.5x10^4^ polarized macrophages were seeded in a final volume of 150 µl TCM. After 72 h, PD-L1 levels on tumor cells and macrophages as well as the phenotype of macrophages were characterized *via* immunofluorescence staining and subsequent flow cytometric analysis. To assess PDAC cell death and the effector phenotype of CD8+ T cells, mono- or co-cultured spheroids were seeded as described above and after 24h spheroids were either left untreated or treated with 10 µg/ml Gemcitabine (Hexal, Holzkirchen, Germany) for further 24h. Afterwards, the medium was exchanged and spheroids either remained untreated or activated CD8+ T cells were added in a 1:10 target/effector cell ratio. In additional settings, spheroids were also treated with 10 µg/ml Durvalumab (Astra Zeneca, Cambridge, UK), Pembrolizumab (Merck/MSD pharma, Rahway, USA), or respective isotype controls (human IgG_1_ or human IgG_4_). After 24 h, supernatants were collected and CD8+ T cells were analyzed *via* flow cytometry.

### 2.8 LEGENDplex

LEGENDplex Human CD8/NK mix and match panel (BioLegend) was used to determine cytotoxic molecules and cytokines (Granzyme A, Granzyme B, Perforin, Granulysin, IFN-γ) in the supernatant of spheroid cultures according to the instructions of the manufacturer. For this purpose, supernatants were centrifuged at 15000xg, 4°C for 8 min to remove cell debris. For analysis, 12.5 µl of the supernatants were used. Measurement was performed using a BD FACSymphony A1 (Beckton Dickinson, East Rutherford, USA) and evaluation was performed with the LEGENDplex-data analysis software v8 (BioLegend).

### 2.9 M30 CytoDeath

For the cell-specific detection of cell death induction in PDAC cells, the M30 CytoDeath™ (PEVIVA^®^, Diapharma, West Chester, USA) ELISA was used. The assay detects caspase-cleaved Keratin 18 (ccK18) which is generated in the present culture settings exclusively by apoptotic PDAC cells. The ELISA was conducted according to the manufacturer’s instructions and measured with a Tecan Infinite^®^ 200 PRO Microplate Reader (Tecan, Männedorf, Switzerland). Resulting values were normalized to the respective PDAC cell count.

### 2.10 Immunofluorescence staining and flow cytometric analyses

Immunofluorescence staining was performed to characterize the phenotype of CD8+ T cells and macrophages as well as PD-L1 surface level of macrophages and PDAC cells. For this purpose, cells were washed with PBS and treated with FcR blocking reagent (human TruStain FcX, Biolegend) diluted 1:10 in MACS buffer (0.5% BSA, 2 mM EDTA in PBS). All following steps were conducted on ice in a 96-well v-bottom plate. After FcR blocking, cells were centrifuged, supernatants were discarded and cells were resuspended in MACS buffer supplemented with respective fluorochrome-conjugated antibodies. All antibodies were purchased from Biolegend. All isotype controls, if not other specified were clone MPOC-2: mIgG1-FITC, mIgG1-APC, mIgG1-PE, mIgG1-PeCy7, mIgG2a-PE (clone MOPC-173), mIgG2b-APC (clone MPC-11), specific antibodies: CD8-FITC (clone HIT8a), CD25-APC (clone BC96), CD69-PE (clone FN50), PD-1-PE (clone EH12.2H7), PD-L1-PeCy7 (clone MIH3), CD163-FITC (clone HGI/61), CD14-PE (clone M5E2), EpCAM-APC (clone 9C4). Cells were stained in antibody solutions for 30 min at 4°C in the dark. Afterwards, cells were washed twice and fixed with 1% PFA in MACS buffer. Immunofluorescence-stained cells were detected by a MACSQuant X flow cytometer and analyzed using the FlowJo software v10.7.1 (Becton Dickinson, Franklin Lakes, USA). Specific immunofluorescence staining was assessed by normalization of the staining intensity of the specific antibody to the staining intensity of the isotype control and is presented as median fluorescence intensity (MFI).

### 2.11 Relative quantification of PDAC cell and macrophage ratios after spheroid co-culture

After 72h spheroid cultivation, supernatants of co-culture samples containing macrophages were collected. Then, PDAC spheroids were dissociated by incubation in PBS supplemented with 1% trypsin (Biowest, Nuaillé, France) for 30 min at 37°C. Enzymatic dissociation was assisted by mechanical dissociation straining the cell suspension through a 30 G cannula every 10 min. Next, macrophage containing supernatants and dissociated respective spheroids were pooled and subjected to immunofluorescence staining and subsequent flow cytometric analysis using a MACSQuant X flow cytometer. For discrimination of PDAC cells and macrophages, cells were stained with EpCAM-APC and CD14-PE antibody, respectively. Analysis was performed using the FlowJo software v10.7.1 (Becton Dickinson, Franklin Lakes, USA).

### 2.12 Statistics

Statistical analysis was performed using GraphPad Prism Version 9.4.1 (GraphPad Software Inc., La Jolla, US). Gaussian distribution of data was tested by Shapiro-Wilk test. In case data sets passed the normality test, a one-way ANOVA followed by either Tukey`s multiple comparison or Dunnett’s multiple comparison (for normalized data sets) test were performed. Parametrically distributed grouped data sets were analyzed by two way-ANOVA followed by multiple comparison Tukey test or Dunnett’s multiple comparison test (for normalized data sets). Parametric data sets are presented by column bar graphs with mean and standard deviation, non-parametric data sets are depicted by bar graphs with median and interquartile ranges in both directions. Results were considered statistically significant for p-values < 0.05. Significance levels are indicated by asterisks: * = *p* < 0.05, **= *p* < 0.01, *** = *p* < 0.001, ****= *p* < 0.0001.

## 3 Results

### 3.1 Spatial expression of PD-L1 in primary PDAC and corresponding liver metastases is associated with the presence of tumor-associated macrophages and CD8+ T cells

**Figure 1:**
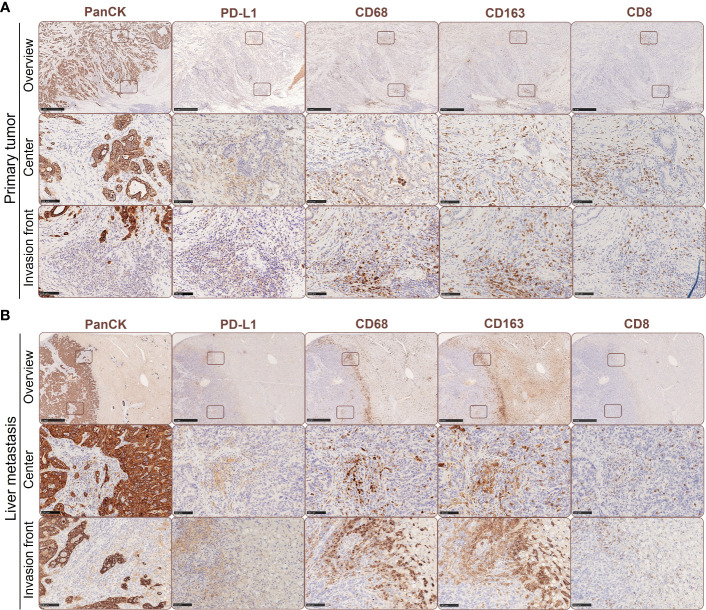
Spatial expression of PD-L1 in primary PDAC and corresponding liver metastases is associated with the presence of tumor-associated macrophages and CD8+ T cells. Representative immunohistochemical stainings for PanCK, PD-L1, CD68, CD163 and CD8 in primary tumors **(A)** and liver metastases of PDAC patients **(B)**. Scale bar overview pictures (above): 1000 µm, areas within tumor or invasion front (below): 100 µm.

**Figure 2:**
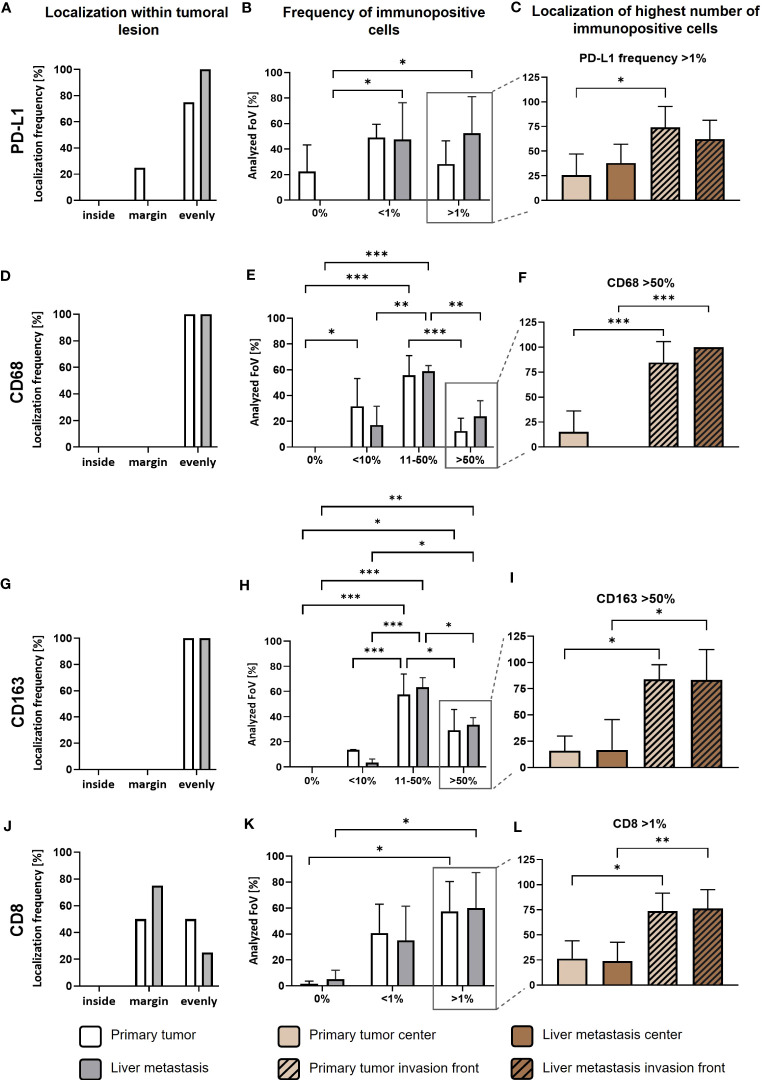
PD-L1 is predominantly expressed in tumor areas characterized by high infiltration of TAMs and CD8+ T cells in primary PDAC tissues and corresponding liver metastases. First, quantitative marker distribution of PD-L1 **(A)**, CD68 **(D)**, CD163 **(G)** and CD8 **(J)** in primary tumors (white) or liver metastases (grey) was assessed by considering localization of immunopositive cells in an overview picture of each tumoral lesion (inside, margin or evenly distributed between inside and margin). Then, percentage of PD-L1 **(B)**, CD68 **(E)**, CD163 **(H)** and CD8 **(K)** immunopositive cells within tumoral lesions was determined. Finally, localization of the highest frequencies (peak category) for PD-L1 **(C)**, CD68 **(F)**, CD163 **(I)** and CD8 **(L)** immunopositive cells was determined. For percentage of immunopositive cells, ten fields of view (FoV) at the invasion front and center (at least one FoV away from invasion front) of the primary tumor or metastasis were evaluated according to the following classification: PD-L1 and CD8: 0%, <1% and >1% immunopositive cells or CD68 and CD163: 0%, <10%, 11-50% or >50% immunopositive cells. Two-way ANOVA with Tukey’s multiple comparison test and One-way ANOVA with Tukey’s multiple comparison test. n=4. * = p<0.05; ** = p<0.01; *** = p<0.001.

As previously reported, PD-L1 is predominantly expressed by stroma cells in primary PDAC (23). In order to examine potential correlations between PD-L1 expression, its spatial distribution and TME alterations during PDAC progression, we performed IHC stainings to detect PD-L1 expression in relation to tumor cells (pan-CK), macrophages (CD68) with an M2-like phenotype (CD163) and CD8+ T cells (CD8) in serial sections from primary tumor (**Figure 1A**) and corresponding liver metastases (**Figure 1B**) of four PDAC patients. We identified PD-L1 expression in all primary PDAC tissues, being evenly distributed within tumor center and the invasion front (**Figures 1A**, **2A**). Of note, all corresponding liver metastases also showed PD-L1 expression with a similar localization pattern (**Figures 1B**, **2A**). However, PD-L1 negative fields of view (FoV) were only observed in primary tumors but not in metastases and a higher proportion of PD-L1 positive FoV (>1% PD-L1+ cells in FoV) was detected in liver metastases (**Figure 2B**). Interestingly, PD-L1 expression was predominantly located at the invasion fronts of both, primary tumors and liver metastases within the group of FoV with highest PD-L1 frequency (<1% PD-L1+) (**Figure 2C**). In line, the frequency of FoV with strong PD-L1 staining intensity was significantly higher in liver metastases compared to primary tumors (**Supplementary Figure 1**). Similar to the expression pattern of PD-L1, CD68+ macrophages were found evenly distributed inside tumoral lesions and at the invasion fronts in both, the primary tumor and liver metastases (**Figures 1A, B**, **2D**). These stainings also demonstrate that areas showing strong PD-L1 staining in primary tumors and liver metastases were characterized by a higher-than-average presence of macrophages, especially CD163+ macrophages (**Figures 1A, B**, **Figures 2E–I**). In over 50% of analyzed FoV, 11-50% of all cells were CD68+ and CD163+ (**Figures 2E, H**). Of note, within the group of FoV that showed the highest frequency of CD68 and CD163+ cells (> 50%), over 50% of these FoV were located at the invasion front of primary or metastatic lesions (**Figures 2F, I**). Similar to this, CD8+ cells were also associated with PD-L1+ areas (**Figures 1A, B**). In primary tumor tissues, CD8+ cells were found in two cases only at the invasion front and in the other two cases CD8+ cells were evenly distributed in the tumor. In liver metastases, CD8+ cells were found in 3 of 4 cases exclusively at the invasion front (**Figure 2J**). In more than 50% of FoV, more than 1% of all analyzed cells were CD8+ (**Figure 2K**). More precisely, most of these FoV enriched for CD8+ cells were located at the invasion front of primary and metastatic lesions (**Figure 2L**). Finally, double IHC stainings of liver tissues revealed that PD-L1 expression was more abundantly colocalized with macrophages (and also hepatic myofibroblasts) than PDAC cells (**Supplementary Figure 2**).

Overall, these data indicate that the presence of PD-L1 in primary PDAC seems to be associated with PD-L1 expression in liver metastases indicating that PD-L1 mediated immune evasion might operate during PDAC progression. As already shown in primary tumors (23, 24), PD-L1 is more frequently expressed by macrophages than PDAC cells and often found at the invasion fronts of tumoral lesions. This spatial PD-L1 expression is associated with the presence of CD8+ T cells, which however are mostly located at the margin/outside the tumoral lesions. Therefore, the question rises whether high PD-L1 expression on macrophages impairs the effector phenotype and killing activity of CD8+ T cells in PDAC.

### 3.2 Co-culture spheroids of PDAC cells and macrophages simulate PD-L1 expression and tumor stroma conditions observed in primary PDAC and liver metastases

**Figure 3:**
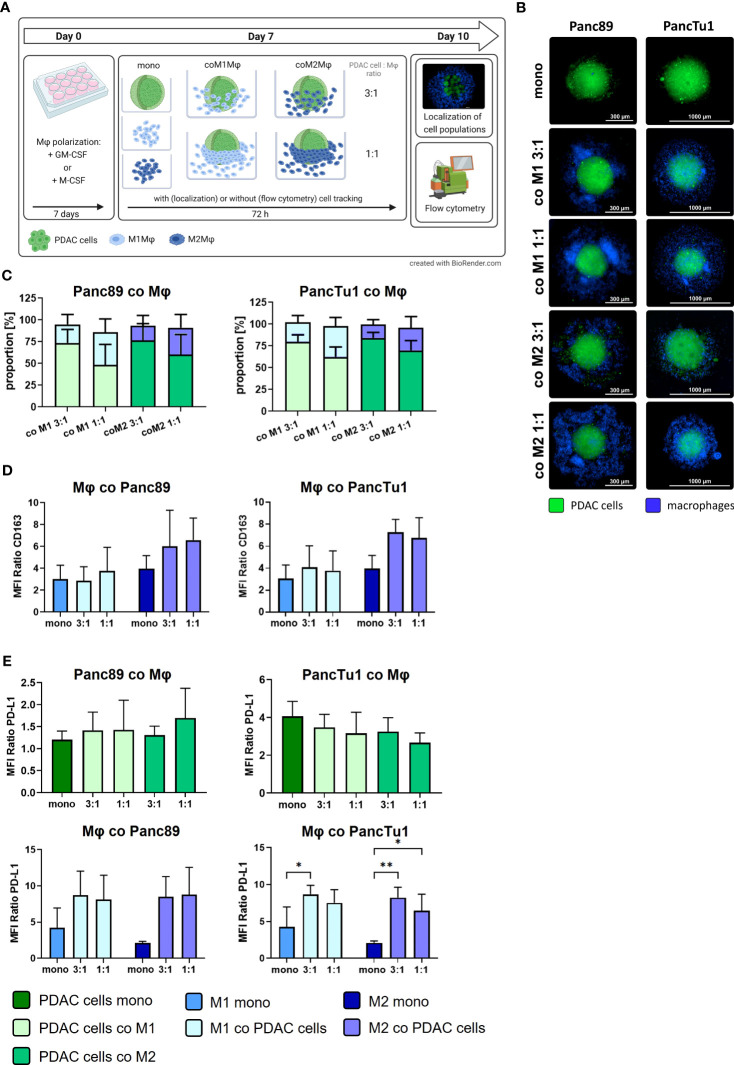
Co-culture spheroids containing PDAC cells and macrophages simulate PD-L1 expression and tumor stroma conditions in primary PDAC and liver metastases. PancTu1 and Panc89 cells were either mono- or co-cultured with M1Mϕ or M2Mϕ at ratios of 3:1 or 1:1 for 72 h in ultra-low attachment plates to form spheroids. **(A)** Schematic illustration of experimental set-up. **(B)** Representative fluorescence microscopy images of CellTracker green labeled Panc89 cells (left) or PancTu1 cells (right) and CellTrace violet labeled Mϕ (violet) at indicated co-culture conditions. (Scale bar PancTu1: 1000 µm; Panc89: 300 µm). **(C)** Proportions of PDAC cells and Mϕ in co-culture 72 h after seeding. To discriminate PDAC cells from Mϕ, single cell suspension of spheroids was stained with EpCAM and CD14-targeting antibodies for flow cytometric analysis (light green: PDAC cells co-cultured with M1Mϕ, dark green: PDAC cells co-cultured with M2Mϕ, light blue: M1Mϕ co-cultured with PDAC cells, dark blue: M2Mϕ co-cultured with PDAC cells). Ratio 3:1: 1.5x10^4^ PDAC cells and 0.5x10^4^ Mϕ; ratio 1:1: 1x10^4^ PDAC cells and 1x10^4^ Mϕ. n=4. **(D)** Relative cell surface expression levels of CD163 on mono- or co-cultured Mϕ. Data is presented as MFI ratio. n=3. **(E)** Relative cell surface expression levels of PD-L1 on mono- or co-cultured Panc89 (left, above) or PancTu1 cells (right, above) as well as Mϕ in co-culture with Panc89 (left, below) or PancTu1 cells (right, below). Data is presented as MFI ratio. Two-way ANOVA with Tukey’s multiple comparison test. n=5 (except mono-cultured Mϕ n=3). * = p<0.05; ** = p<0.01.

To simulate the TME conditions in primary PDAC and liver metastases identified *in situ*, we set-up a PDAC co-culture spheroid model comprising PDAC cells with different status of driver mutations and PD-L1 expression as well as different ratios and types of macrophages to examine expression and role of PD-L1 in immune evasion of PDAC cells. During PDAC progression, the number of macrophages increases with the stage of disease (32) and a higher tumor infiltration by CD163+ macrophages correlates with shorter 5-year overall and recurrence-free survival (33). Therefore, we used a PDAC cell to macrophage ratio of 3:1 to mimic an infiltration by macrophages at primary site, whereas 1:1 ratio served as model for macrophage infiltration into liver metastasis. **Figure 3A** shows a schematic illustration of the experimental setup and **Figure 3B** depicts representative images of mono- and co-cultured spheroids of Panc89 and PancTu1 PDAC cells. After 72 h, both PDAC cell lines formed dense tumor spheroids and in co-cultured spheroids, macrophages were mostly located at the spheroid margin, being more pronounced in spheroids with higher amounts of macrophages (1:1). Flow cytometric analysis of PDAC cells and macrophages revealed that in co-culture spheroids the proportion of PDAC cells and macrophages remained almost stable over culture duration (**Figure 3C**). Next, it was investigated whether the presence of PDAC cells impacts macrophage polarization as macrophages exhibit a high plasticity and can change their phenotype due to surrounding microenvironment (6, 34, 35). Mono-cultured M2-like macrophages exhibited higher CD163 surface levels compared to M1-like macrophages. After 72 h co-culture with Panc89 or PancTu1 cells at either cell ratio, CD163 expression on M1-like macrophages was hardly affected compared to mono-cultured cells. In contrast, M2-like macrophages exhibited intensified CD163 surface levels after co-culture with either PDAC cell line (**Figure 3D**). These findings indicate that proportions of PDAC cells and macrophages as well as macrophage phenotypes remained stable in co-culture spheroids over a culture duration of 72h.

Furthermore, PD-L1 surface levels were analyzed in mono- and co-cultured Panc89 and PancTu1 cells as well as macrophages (**Figure 3E**). Mono-cultured Panc89 cells exhibited hardly any PD-L1 surface expression and this was almost not affected by either co-culture with macrophages (green bars, MFI ratio 1.2-1.69). Contrastingly, mono-cultured PancTu1 cells were characterized by considerable PD-L1 surface levels (dark green bar, MFI ratio 4.05), which, however, were not further increased by macrophages in either co-culture setting. In contrast, PD-L1 surface levels were detected at higher levels in both mono-cultured macrophage populations, albeit M1-like macrophages demonstrated higher expression levels compared to M2-like macrophages (blue bars, MFI ratio M1-like versus M2-like macrophages: 4.22 versus 2.1). Of note, co-culture with both PDAC cell lines clearly increased PD-L1 surface levels in both macrophage populations up to a similar level even if lower proportions of PDAC cells were present in the spheroids (1:1) (**Figure 3E**).

Overall, these results demonstrate that we established a stable PDAC cell spheroid model enriched with M1- or M2-like macrophages, that well reflects both cellular composition as well as PD-L1 expression levels identified in our *in situ* analyses of primary PDAC and liver metastases tissues. Moreover, higher PD-L1 expression was observed in macrophages rather than PDAC cells, being also in line with our *in situ* findings. Of note, the presence of PDAC cells further increased PD-L1 surface levels in either macrophage population.

### 3.3 PDAC spheroids comprising PD-L1 expressing M1- or M2-like polarized macrophages do not impair the effector phenotype of CD8+ T cells which is not associated with PDAC cell death induction

**Figure 4:**
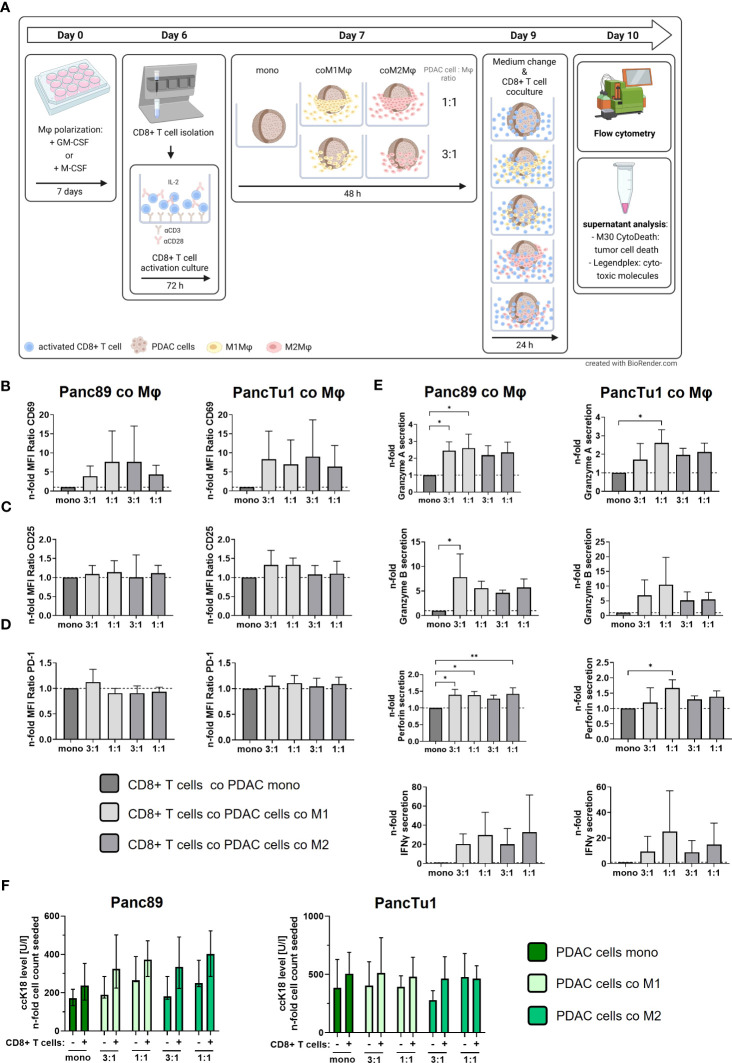
PDAC spheroids comprising PD-L1 expressing M1- or M2-like polarized macrophages do not impair the effector phenotype of CD8+ T cells but which is no associated with PDAC cell death. PancTu1 or Panc89 cells were mono- or co-cultured with either M1- or M2-like polarized Mϕ in ultra-low attachment plates to form spheroids. After 48 h, pre-activated CD8+ T cells were added to spheroids for 24 h. **(A)** Schematic illustration of experimental set-up. Immunofluorescence staining of activation markers **(B)** CD69, **(C)** CD25 and **(D)** PD-1 **(D)** on CD8+ T cells cultured with either mono-cultured Panc89 (left panel) or PancTu1 cells (right panel) or with the indicated co-cultures with M1- (light grey) or M2Mϕ (dark grey), measured *via* flow cytometer. Relative cell surface expression levels are normalized to the respective culture of CD8+ T cells with mono-cultured Panc89 or PancTu1 spheroids. Data is expressed as n-fold MFI ratio of specific staining. n=4. **(E)** Granzyme A, Granzyme B, Perforin and IFNγ concentration in supernatants of CD8+ T cells cultured with either mono-cultured Panc89 (left panel) or PancTu1 spheroids (right panel) or with the indicated co-culture spheroids with M1- (light grey) or M2Mϕ (dark grey). Concentrations were measured by multiplex assay and data is normalized to the respective cytokine concentrations of CD8+ T cells in culture with mono-cultured PDAC spheroids. One-way ANOVA using Dunnett’s multiple comparison test comparing all samples with a control of 1. The dashed lines mark a concentration ratio of “1”. n=3. **(F)** Supernatant levels of caspase-cleaved Keratin 18 (ccK18) in mono- or co-culture spheroids of Panc89 (left) or PancTu1 cells (right) and Mϕ without **(-)** or with CD8+ T cells (+). ccK18 levels were normalized to the respective PDAC cell count. Mono: 2x10^4^ PDAC cells; ratio 3:1: 1.5x10^4^ PDAC cells and 0.5x10^4^ Mϕ; ratio 1:1: 1x10^4^ PDAC cells and 1x10^4^ Mϕ. Not normally distributed data are depicted as median with interquartile range in both directions. Panc89: n=4; PancTu1: n=3. * = p<0.05.

Next, we examined whether PD-L1 expressing macrophages in the different co-culture spheroids impact the effector phenotype and killing activity of CD8+ T cells. For this purpose, pre-activated CD8+ T cells were added to the mono- and co-culture spheroids (**Figure 4A**). Notably, after activation culture these pre-activated CD8+ T cells exhibited high cell surface expression levels of CD25 and the early activation marker CD69 along with considerable cell surface levels of PD-1 (**Supplementary Figures 3A-C**), pointing to an exhausted T cell phenotype often found in PDAC tissues (36–38). First, we investigated whether cell surface expression levels of CD69 and CD25 of pre-activated CD8+ T cells are altered by the different spheroid co-culture conditions. In comparison to mono-cultured pre-activated CD8+ T cells, CD8+ T cells cultured with PDAC mono-culture spheroids did not alter their CD69, CD25 and PD-1 surface expression (**Supplementary Figures 4A-C**). However, we observed that CD69 expression levels on CD8+ T cells were enhanced by trend after all macrophage co-culture conditions with both PDAC cell lines (**Figure 4B**), albeit its induction was more pronounced after co-culture with PancTu1 spheroids (n-fold MFI ratio 6,36 – 9.02) compared to Panc89 spheroids (n-fold MFI ratio 3.86 –7.68). Interestingly, co-culture with different ratios or polarizations of macrophages did not affect these elevated CD69 cell surface levels on CD8+ T cells. CD25 cell surface levels of CD8+ T cells were almost not affected by the different PDAC spheroid conditions (**Figure 4C**). Since PD-L1 cell surface expression levels were increased in macrophages after co-culture in PDAC spheroids (**Figure 3E**), we next examined whether PD-1 cell surface expression levels are altered in CD8+ T cells after the different co-culture conditions. However, PD-1 cell surface levels on CD8+ T cells were not affected by any spheroid co-culture setting (n-fold MFI ratio 0.91 – 1.12, **Figure 4D**).

Besides the expression of activation-associated marker proteins, the secretion of cytokines and cytotoxic effector molecules represents a hallmark of T cell activation and effector function. Therefore, we analyzed the levels of Granzyme A/B, Perforin and IFNγ in supernatants obtained from CD8+ T cells either cultured alone or cultured with mono- or co-culture spheroids. Compared to pre-activated mono-cultured CD8+ T cells, concentrations of Granzyme A and B, Perforin and IFNγ were clearly enhanced in supernatants of pre-activated CD8+ T cells in presence of mono-culture PDAC spheroids, an effect which was even more pronounced in culture with mono-culture PancTu1 spheroids (**Supplementary Figures 5A-D**). Thus, the presence of PDAC cells increased release of CD8+ T cell effector molecules. However, even more elevated levels of Granzyme A and B, Perforin as well as IFNγ (by trend) were detectable in supernatants derived from CD8+ T cells cultured with macrophage enriched PDAC spheroids compared to supernatants from CD8+ T cells cultured with mono-culture PDAC spheroids (**Figure 4E**). Overall, higher levels of all effector molecules were detected in supernatants of CD8+ T cells and co-culture spheroids containing an equal number of PDAC cells and macrophages, particularly M1-like polarized macrophages (1:1). Finally, we analyzed whether the observed alterations of the T cell effector phenotype correlate with PDAC cell death induction under the different stroma conditions. PDAC cell death was assessed by determination of an effector caspase-cleaved Keratin 18 fragment (ccK18) released in supernatants, indicative for epithelial cells undergoing apoptotic cell death (**Figure 4F**). Slightly increased ccK18 levels were detected in supernatants derived from CD8+ T cells cultured with mono- or co-cultured PDAC cell spheroids compared to supernatants from respective spheroid culture without CD8+ T cells (ΔccK18 level: Panc89 median (range): 136 (67-153), PancTu1 median (range): 122 (87-184)). However, no considerable effect by the different co-culture settings was observed.

In summary, these data indicate that the effector phenotype of CD8+ T cells exemplified by expression activation markers and release of effector molecules is not impaired in the presence of PD-L1 expressing macrophages particularly with an M1-like phenotype. In contrast, our findings rather suggest that the effector phenotype of CD8+ T cells is maintained or even promoted in presence of macrophages and PDAC cells independent of their PD-L1 expression, but which is not associated with increased PDAC cell death.

### 3.4 Blocking of PD-1 and PD-L1 does not enhance the effector phenotype of CD8+ T cells and does not increase PDAC cell death

**Figure 5:**
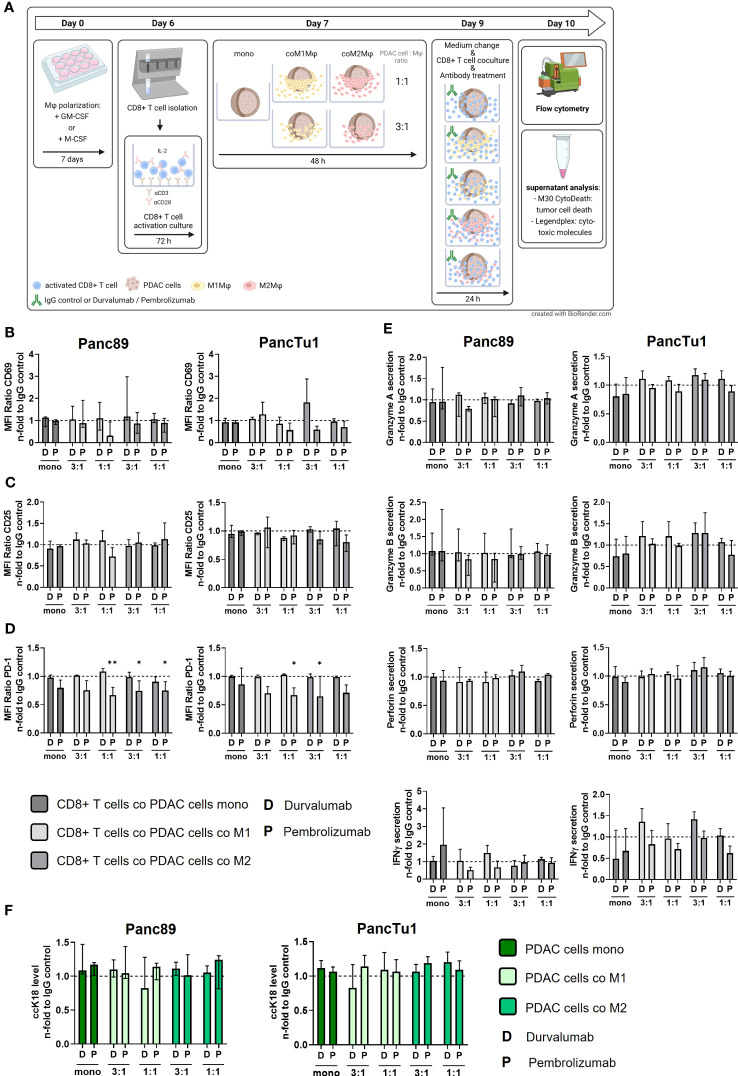
Blocking of PD-1 and PD-L1 only slightly affects the effector phenotype of CD8+ T cells and does not increase PDAC cell death. PancTu1 or Panc89 cells were mono- or co-cultured with either M1- or M2-like polarized Mϕ in ultra-low attachment plates to form spheroids. After 48 h, medium was changed, mono- or co-cultures were treated with either Durvalumab (D) or Pembrolizumab (P) or their respective isotype controls (10 µg/ml) and CD8+ T cells were added for 24 h. **(A)** Schematic illustration of experimental set-up. Immunofluorescence staining of activation marker **(B)** CD69, **(C)** CD25 and **(D)** PD-1 on CD8+ T cells cultured with mono-cultured PDAC cell spheroids (dark grey) or co-cultured Panc89 (left panel) or PancTu1 (right panel) cell spheroids with M1Mϕ (light grey) or M2Mϕ (middle grey). Immunofluorescence staining was measured by flow cytometer and data is normalized to the respective IgG control samples of the indicated culture conditions. **(E)** Granzyme A, Granzyme B, Perforin and IFNγ concentration of supernatants of CD8+ T cells cultured with either mono-cultured (dark grey) Panc89 (left panel) or PancTu1 spheroids (right panel) or with the indicated co-cultures with M1- (light grey) or M2Mϕ (middle grey) spheroids. Concentrations were measured by multiplex assay and data was normalized to the respective IgG control samples of the indicated culture conditions. **(F)** Levels of caspase-cleaved Keratin 18 (ccK18) in supernatants of CD8+ T cells cultured with mono-culture spheroids of Panc89 (left) or PancTu1 cells (right) or with the indicated Mϕ co-culture conditions. ccK18 levels were normalized to the respective PDAC cell count and then to the respective IgG control samples of the indicated co-culture conditions. Mono: 2x10^4,^ratio 3:1: 1.5x10^4^ PDAC cells and 0.5x10^4^ Mϕ; ratio 1:1: 1x10^4^ PDAC cells and 1x10^4^ Mϕ. Not normally distributed data are depicted as median with interquartile range in both directions. Two-way ANOVA with Dunnett’s multiple comparison test comparing all samples with a control of 1. The dashed lines mark an MFI ratio of “1”. n=3. * = p<0.05.

Based on the fact that both macrophage populations as well as PancTu1 cells exhibit considerable PD-L1 expression (**Figure 3E**) and CD8+ T cells show marked PD-1 expression (**Supplementary Figure 3**), we next investigated whether antibody mediated blockade of PD-L1 (by Durvalumab) or PD-1 (by Pembrolizumab) impacts the effector phenotype of CD8+ T cells leading to increased PDAC cell death. **Figure 5A** shows a schematic illustration of the experimental set-up. Durvalumab treatment did neither impact CD69 expression (**Figure 5B**) nor CD25 expression (**Figure 5C**) of CD8+ T cells cultured under either mono- or co-culture PDAC spheroid conditions. Furthermore, PD-1 surface levels were not changed after Durvalumab treatment in comparison to respective IgG controls in all co-culture conditions with each PDAC cell line, but were significantly decreased on CD8+ T cells derived from all co-culture settings treated with Pembrolizumab (**Figure 5D**). This finding can be explained by the fact that Pembrolizumab bound to PD-1 blocks the PD-1 epitope recognized by the staining antibody. Therefore, these results confirmed successful inhibition of PD-1 in our experimental set-up. Successful inhibition of PD-L1 by Durvalumab could also be confirmed as PD-L1 was not detectable in Durvalumab treated macrophages and PDAC cells in all co-culture (**Supplementary Figure 6**). Next, the levels of cytotoxic effector molecules and IFNγ were investigated in supernatants from the different Durvalumab or Pembrolizumab treated spheroids. Overall, neither Granzyme A and B nor Perforin levels in supernatants of CD8+ T cells cultured with either mono- or co-culture spheroids were altered after Durvalumab or Pembrolizumab treatment (**Figure 5E**). Durvalumab treatment led to slightly increased secretion of IFNγ by trend into supernatants of CD8+ T cells co-cultured with PancTu1 spheroids at a ratio of 3:1 with either macrophage population, while Pembrolizumab slightly diminished IFNγ secretion under almost all co-culture conditions (**Figure 5E**). Finally, ccK18 levels of mono- and macrophage enriched PDAC cell spheroids after culture with CD8+ T cells and ICI treatment were measured in order to assess PDAC cell death induction under treatment of the different stromal conditions. In line with the findings regarding the effector phenotype of CD8+ T cells, no considerable effects were observed on PDAC cell death by either antibody treatment in all PDAC cell spheroid cultures (**Figure 5F**).

In summary, neither Durvalumab nor Pembrolizumab treatment impacted the effector phenotype of CD8+ T cells after co-culture with any PDAC cell spheroids and macrophages ratios. Moreover, none improvement of tumor cell death induction in both PDAC cell spheroid models was detected independently of the amount and subtype of macrophages and treatment with either Durvalumab or Pembrolizumab.

### 3.5 Sequential treatment with Gemcitabine and immune checkpoint blockade does not lead to increased PDAC cell death

**Figure 6:**
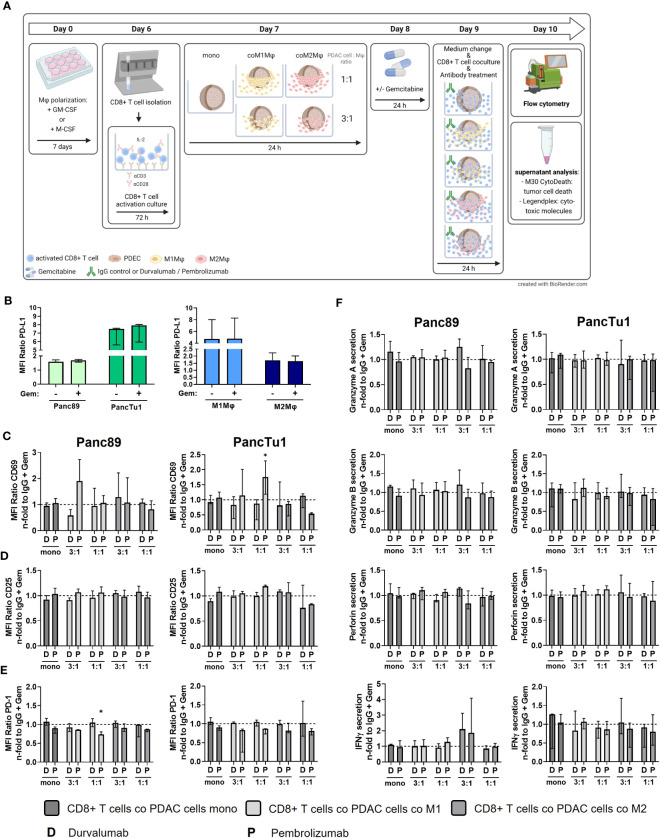
Sequential treatment with Gemcitabine and Durvalumab or Pembrolizumab does not improve CD8+ T cell effector phenotype. PancTu1 or Panc89 cells were mono- or co-cultured with either M1- or M2-like Mϕ for 24 h in ultra-low attachment plates to form spheroids. After 24 h, mono- or co-cultures were treated with Gemcitabine (Gem) (10 µg/ml) for 24 h. Afterwards, medium was changed, and cultures were treated with either Durvalumab (D) or Pembrolizumab (P) or their respective isotype controls (10 µg/ml) and simultaneous CD8+ T cell co-culture was started for 24 h. **(A)** Schematic illustration of experimental set-up. **(B)** PD-L1 staining of mono-cultured Panc89 or PancTu1 cells as well as M1- or M2-like Mϕ which were left untreated **(-)** or treated with Gemcitabine (+) for 24 h PD-L1 cell surface level was measured by flow cytometer and data is depicted as MFI ratio of specific staining. Immunofluorescence staining of activation marker **(C)** CD69, **(D)** CD25 and **(E)** PD-1 on cells surfaces of CD8+ T cells cultured with mono-cultured PDAC cell spheroids (dark grey) or co-cultured Panc89 (left panel) or PancTu1 (right panel) spheroids with M1Mϕ (light grey) or M2Mϕ (middle grey) after sequential treatment with Gemcitabine and ICI. Cell surface levels were measured by flow cytometer and data was normalized to the respective IgG control treated cells of the indicated Gemcitabine treated culture conditions. **(F)** Detection of Granzyme A, Granzyme B, Perforin and IFNγ in supernatants of CD8+ T cells cultured with either mono-cultured (dark grey) Panc89 (left panel) or PancTu1 spheroids (right panel) or with the indicated co-cultures with M1- (light grey) or M2Mϕ (middle grey) spheroids after sequential treatment with Gemcitabine and ICI. Concentrations were measured by multiplex assay and data is normalized to the respective IgG control samples of Gemcitabine treated culture conditions. Mono: 2x10^4^, ratio 3:1: 1.5x10^4^ PDAC cells and 0.5x10^4^ Mϕ; ratio 1:1: 1x10^4^ PDAC cells and 1x10^4^ Mϕ. Not normally distributed data are depicted as median with interquartile range in both directions. Two-way ANOVA with Dunnett’s multiple comparison test comparing all samples with a control of 1. The dashed lines mark an MFI ratio of “1”. * = p<0.05. n=3.

**Figure 7:**
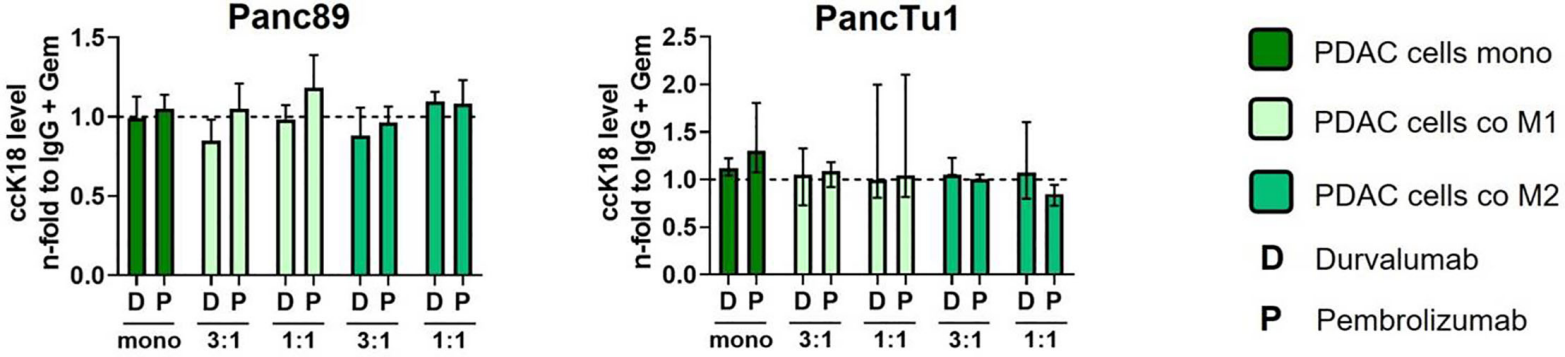
Sequential treatment with Gemcitabine and immune checkpoint blockade does not lead to increased PDAC cell death. PancTu1 or Panc89 cells were mono- or co-cultured with either M1- or M2-like Mϕ for 24 h in ultra-low attachment plates to form spheroids. After 24 h, mono- or co-cultures were treated with Gemcitabine (Gem) (10 µg/ml) for 24 h. Afterwards, medium was changed, and cultures were treated with either Durvalumab (D) or Pembrolizumab (P) or their respective isotype controls (10 µg/ml) and simultaneous CD8+ T cell co-culture was started for 24 h. Levels of caspase-cleaved Keratin 18 (ccK18) in supernatants of CD8+ T cells cultured with mono-culture spheroids of Panc89 (left) or PancTu1 cells (right) and with the indicated Mϕ co-culture conditions after sequential treatment with Gemcitabine and ICI. ccK18 levels were normalized to the respective IgG control samples of the indicated Gemcitabine treated culture conditions. Mono: 2x10^4,^ratio 3:1: 1.5x10^4^ PDAC cells and 0.5x10^4^ Mϕ; ratio 1:1: 1x10^4^ PDAC cells and 1x10^4^ Mϕ. Not normally distributed data are depicted as median with interquartile range in both directions. Two-way ANOVA with Dunnett’s multiple comparison test comparing all samples with a control of 1. The dashed lines mark an MFI ratio of “1”.

Standard chemotherapy agents can alter the expression of tumor-specific membrane antigens which can result in better antigen presentation *via* antigen-presenting cells and more effective cytotoxic T lymphocytes response (39) Thus, the modulation of tumor-specific membrane proteins as well the enhancement of T cell infiltration by (low dose) cytostatic drugs may serve as a promising strategy to improve the immunotherapy (39, 40). Furthermore, as most PDAC patients are treated with cytostatic drugs as first-line therapies (41), we investigated whether sequential therapy of low dose Gemcitabine followed by immune checkpoint blockade modulates the effector phenotype of CD8+ T cells and leads to elevated induction of PDAC cell death. For this purpose, mono- and macrophage co-cultured Panc89 and PancTu1 spheroids were treated with Gemcitabine for 24 h and then cultured with pre-activated CD8+ T cells and treated with Durvalumab, Pembrolizumab or respective isotype control (**Figure 6A**). Gemcitabine treatment led to slightly elevated ccK18 levels in supernatants of Panc89 spheroids and rather diminished levels in those of PancTu1 spheroids. However, overall Gemcitabine treatment hardly modulated ccK18 levels in supernatants of mono- and co-cultured PDAC spheroids, indicating that this dose does not lead to massive PDAC cell death under these conditions (**Supplementary Figure 7A**). Furthermore, Gemcitabine treatment did not impact PD-L1 expression of PDAC cells and either macrophage population (**Figure 6B**). To analyze whether Gemcitabine pretreatment of PDAC spheroids improves the efficacy of ICI treatment, the effector phenotype of CD8+ T cells and PDAC cell death was assessed compared to ICI treatment alone. To specifically outline the effects exerted by combined treatment, data shown in **Figures 6C–F** and **7** were derived only from Gemcitabine pretreated cultures with either additional treatment with ICI or isotype control antibody. Analyzing activation markers in CD8+ T cells under the different culture conditions revealed that combined treatment with Pembrolizumab enhanced CD69 surface levels in CD8+ T cells after culture with M1-like macrophage enriched spheroids of both PDAC cell lines, while it led to reduced CD69 levels in CD8+ T cells derived from PancTu1 spheroids comprising M2-like macrophages (1:1). In contrast, combined treatment with Durvalumab hardly impacted CD69 levels of CD8+ T cells cultured with either mono- or co-cultured PDAC spheroids (**Figure 6C**). No clear effects were detected for CD25 cell surface levels after either treatment (**Figure 6D**). PD-1 cell surface levels of CD8+ T cells were not altered after sequential treatment with Gemcitabine and Durvalumab after all culture conditions, while it was again reduced after treatment with Pembrolizumab (**Figure 6E**). In line with the marginal effects on the activation markers, release of Granzyme A and B, Perforin and IFNγ into supernatants of co-cultured CD8+ T cells was also hardly affected by sequential treatment with Gemcitabine and either immune checkpoint inhibitor (**Figure 6F**). Finally, PDAC cell death was not considerably increased after the different treatments reflected by the almost unaltered ccK18 levels measured in supernatants of the different spheroid cultures (**Figure 7**), while CD8+ T cell culture with either Gemcitabine pretreated PDAC cell spheroids resulted in slightly enhanced ccK18 levels (**Supplementary Figure 7**).

Overall, these data indicate that sequential treatment of PDAC with Gemcitabine and immune checkpoint inhibitors does not improve the effector phenotype of CD8+ T cells and PDAC cell death irrespective of the presence and subtype of macrophages.

## 4 Discussion

PDAC is mostly diagnosed at late stages of the disease due to the lack of specific symptoms and poor specificity of tumor markers. Further, it often metastasizes, especially to distant organs including liver (76-80% of patients), peritoneum (48%) or lung (45%) (42). To date, the only curative treatment option is resection but most of the patients are not eligible due to the late diagnosis or comorbidities. Even if the primary tumor is successfully surgical resected, the 5-year survival rate of these patients is only 27% (43). Therefore, improved therapeutic treatments are urgently needed. Several studies identified expression of the immune checkpoint molecule PD-L1 in PDAC patients (20–24). In patients without metastatic spread, most of these PD-L1 expressing cells are stromal cells (23). While ICI like Durvalumab and Pembrolizumab revolutionized the treatment of many solid malignancies even at advanced stages, e.g. NSCLC (44), single agent use of Durvalumab as well as other ICIs widely failed in PDAC (6, 19). Since the ICI failure in PDAC is still an unresolved issue, and given the role of CD163+ M2-like macrophages as a prominent cell population in the TME of PDAC (10, 24, 45), a better understanding of how stromal macrophages impact the PD-1/PD-L1 axis and activity of CD8+ T cells in this tumor entity is greatly needed.

To unveil whether PD-L1-mediated immune evasion might operate during PDAC progression, we first analyzed serial PDAC tumor tissues and their corresponding liver metastases concerning PD-L1 frequency and intensity as well as the abundance of macrophages and CD8+ T cells. A study by Rahn et al. already reported that in 69.6% of analyzed primary PDAC tissues (all stages T3N1M0) no or only low PD-L1 expression was detectable (23). In contrast, in our small collective of four PDAC patients all primary tumors as well as their corresponding liver metastases showed considerable PD-L1 expression. The higher frequency of PD-L1 expressing primary tumors in the present cohort might be explained by the fact that these patients mostly exhibited poorly differentiated tumors. The observation that poorly differentiated primary PDAC tumors express higher PD-L1 levels was also outlined in a previous study of Rahn et al. (24). Moreover, the fact that PD-L1 is expressed in the primary context as well as in metastases might indicate that PD-L1-mediated immune evasion is important for malignant progression and metastasis of PDAC. Interestingly, our histological analyses of PDAC tissues also revealed that PD-L1 expression was predominantly located in the TME at the tumor invasion fronts along with a high proportion of macrophages (CD68+ and CD163+ cells) supporting the view that the main source of PD-L1 are stromal cells. Double IHC stainings confirmed that PD-L1 is expressed to a much higher extent by macrophages than PDAC cells. This finding is in line with a former study demonstrating that in primary PDAC 76.5% of PD-L1 expressing cells are stromal cells (23). CD8+ T cells could be detected at considerable frequencies in primary PDAC and in liver metastases, however, these cells were mainly located at the invasion fronts of the primary tumor or metastasis and thus separated from the tumor cells by PD-L1 expressing cells (macrophages). In another study analyzing biopsies of liver metastases from PDAC patients, metastatic tumor cells were also surrounded by CD68+ macrophages which was the most prominent immune cell population (46), underlining the results of our study. Moreover, it is known that M2 TAM can suppress immunotherapeutic efficacy of PD-1/PD-L1 inhibition by suppression of T cell activity and enhanced expression of PD-L1 in the TME. Particularly, the secretion of anti-inflammatory cytokines and exosomes can induce expression of immune checkpoint molecules (47). Our observation that especially macrophages are predominantly located at invasion fronts of primary PDAC as well as liver metastasis suggests a potent barrier impairing infiltration and tumor directed immunity of CD8+ T cells. Even taking into account the small size of the tissue cohort studied, the first comparative analysis of primary tumor and corresponding metastases from PDAC patients provides a unique insight into the potential role of PD-L1 and macrophages in immune evasion of PDAC.

To simulate the TME conditions in primary PDAC and liver metastases identified *in situ*, we established two PDAC co-culture spheroid models with different ratios and types of macrophages in order to investigate whether the presence and amount of different types of macrophages impact PD-L1 expression in PDAC cells as well as CD8+ T cell activity. Our 3D co-culture model well simulated the TME conditions detected *in situ*, as macrophages were mostly located at high frequencies at the margin of tumor spheroids. Additionally, the M2-like phenotype of macrophages, indicated by expression of CD163, was further promoted by both PDAC cell spheroid co-cultures. This observation is supported by several studies reporting high abundance of M2 macrophages in the TME of human and murine PDAC (8, 24, 48). Moreover, either macrophage population exhibited considerable PD-L1 expression being in line with our *in situ* analysis which revealed PD-L1 expression more on stromal cells/macrophages rather than tumor cells. Accordingly, we chose PancTu1 cells exhibiting moderate PD-L1 expression and Panc89 cells lacking PD-L1 expression as PDAC cell models for our *in vitro* studies in order to reflect tumor heterogeneity regarding PD-L1 expression in PDAC. While PD-L1 expression was not altered in both PDAC cell lines due to the attendance of either macrophage subtype, it was clearly enhanced in both macrophage populations after co-culture with either PDAC cell line. In contrast to our results, Xia et al. showed a PD-L1 increase on PDAC cells due to the attendance of M2-like macrophages (21). One explanation for this discrepancy might be that in this study a higher macrophage to PDAC cell ratio was used. Moreover, they identified Transforming growth factor-beta1 (TGF-β1) as a main inducer of PD-L1 expression (21), which seemed not be the inducer of PD-L1 expression in our model system, as TGF-β1 was not detectable or only in small amounts in the culture supernatants (data not shown). Thus, our findings support the view that macrophages are a superior source of PD-L1 expression in PDAC, however, the identification of the inducing factors in our PDAC 3D model system is subject of future investigation.

Next, it was investigated whether the attendance of PD-L1 expressing macrophages impacts the effector phenotype of CD8+ T cells. Interestingly, the effector phenotype of CD8+ T cells was rather promoted by either co-culture with macrophages reflected by an increase of the early activation marker CD69 and the release of Granzyme A/B, Perforin and IFNγ. Of note, the release of effector molecules was more increased in the presence of higher number of (M1) macrophages indicating a relationship between the T cell effector phenotype and the abundance of macrophages. In PDAC patients, a high density of CD8+ T cells in tumor areas is associated with better survival outcome (49, 50). Further, several therapy approaches targeting phenotype switch from M2 to M1 macrophages and thereby induce CD8+ T cell response, T cell recruitment and IFN responses (4, 51, 52) underlie our results of enhanced CD8+ T cell effector phenotype in presence of M1 macrophages. Although macrophages showed higher PD-L1 expression in co-culture with PDAC cells, the effector phenotype of CD8+ T cells was rather intensified, but which was not associated with elevated PDAC cell death. Here, it can be speculated whether infiltration of CD8+ T cells into the spheroids is impaired not allowing a further increase in PDAC cell death induction. To this end, we cannot make any reliable conclusion on this issue because our available imaging modalities which do not properly decipher whether CD8+ T cells are infiltrated into the spheroids or whether they are attached at the margin. However, the latter would be in line with our findings *in situ* where CD8+ T cells were also separated from the tumor cells. As high tumoral PD-L1 expression is correlated with poor survival of PDAC patients (21, 53), the concept of inhibiting the PD-1/PD-L1 axis to enhance T cell cytotoxicity is promising. However, single agent immunotherapy with Durvalumab or Pembrolizumab has still failed in clinical trials for PDAC (6). In order to elucidate whether different ratios and phenotypes of macrophages may impact treatment efficacy of Durvalumab or Pembrolizumab, we treated mono- and co-culture spheroids with either antibody. Despite considerable expression of PD-L1 and PD-1 in macrophages and CD8+ T cells, respectively, ICI treatment did not lead to enhanced PDAC cell death in either mono- or co-culture model. These findings are in line with the above-mentioned clinical situation. Another determinant for ICI responses is the tumor mutational burden (TMB). In other malignancies including melanoma, non-small-cell lung cancer and urothelial cell carcinoma, patients with high TMB clearly more benefit from ICI therapy compared to patients with low TMB (54). One explanation for this is that tumors with high TMB exhibit more immunogenic neoantigens, which can be recognized by T cells thereby fostering ICI therapy (54). In PDAC, only 1.1% of patients show a high TMB, supporting the low response rate towards ICI treatment. However, preliminary results of anti-PD-1 treatment in the subgroup of PDAC patients with high TMB revealed promising effects (55). As monotreatment with Durvalumab and Pembrolizumab failed in our 3D co-culture model and Gemcitabine has been identified as inducer of PD-L1 expression (56, 57), we investigated whether sequential treatment with Gemcitabine and ICI showed superior effects on the effector phenotype of CD8+ T cells and induction of PDAC cell death. This concept was further supported by data from a murine model of pancreatic cancer liver metastasis, where the combination of Gemcitabine treatment and anti-PD-1 antibody was associated with infiltration of CD8+ T cells and M1 macrophages along with prolonged survival of the mice (58). However, Gemcitabine treatment did not increase PD-L1 expression in PDAC cells and the combination of Gemcitabine pretreatment and ICI did neither impact the CD8+ T cell activation status nor increased PDAC cell death. Nevertheless, several clinical studies evaluating different combinational treatments are ongoing, e.g., an open label, single arm, multicenter clinical trial investigates the combination of AZD0171 and Durvalumab as well as standard-of-care chemotherapy in metastatic PDAC (NCT04999969). Another randomized multicenter phase Ib/II clinical trial study investigates efficacy of a combination of neoadjuvant chemoradiation therapy with Pembrolizumab treatment in PDAC (NCT02305186).

Besides the PD-L1/PD-1 axis several other mechanisms might operate in PDAC impairing CD8+ T cell mediated tumor reactivity and limiting the effects of PD-1/PD-L1 blockade. Although PD-1/PD-L1 axis is a major player in regulating T cell functions and was efficiently blocked in our *in vitro* experiments, several other co-inhibitory interactions can restrain the anti-tumor function of CD8+ T cells in the TME (59). These co-inhibitory molecules include Inducible T cell costimulator (ICOS), T cell immunoreceptor with Ig and ITIM domains (TIGIT), PD-1 and lymphocyte-activation gene 3 *(*LAG3) were identified at elevated levels on CD8+ T cells in PDAC tissues (60), suggesting that the TIGIT-CD155 or LAG3-MHC interaction are more potent immunosuppressive mechanisms in PDAC. Especially TIGIT, which is expressed on tumor-infiltrating cytotoxic T cells in multiple malignancies and its ligand CD155, often expressed by tumor-infiltrating myeloid cells and upregulated on cancer cells, provide a promising target to overcome the local suppression of immune surveillance. As CD155-TIGIT interaction is associated with cancer resistance to PD-1 blockade, targeting this interaction might be a promising strategy to increase the efficacy of PD-1 inhibition (59). Accordingly, Pearce et al. could recently show that CD8+ tissue-resident memory T cells in PDAC patients co-express PD-1 and TIGIT on their surfaces and blocking of both molecules enhanced IFNγ secretion and T cell proliferation, suggesting a promising route to improve ICI efficacy (61). In line with this hypothesis, Freed-Pastor et al. showed that targeting the TIGIT-CD155 axis in combination with CD40 agonists and anti-PD-1 treatment elicits profound anti-tumor responses in pancreatic cancer mouse models *in vivo* (62). Furthermore, Gulhati et al. demonstrated an enhanced expression of *LAG3, Tnfrsf9 (*41BB) and *Havcr2* (TIM3) in genetically engineered PDAC mouse model under anti-PD-1 monotherapy (48), supporting the view that combined targeting of different immune regulatory mechanisms might be an efficient strategy to induce a potent tumor directed immunity in PDAC. Preclinical and clinical studies underscore this hypothesis as co-blockade of PD-1 and a second co-inhibitory molecule such as CTLA-4 augment the antitumor immunity compared to single PD-1 blockade in different solid malignancies (59). Taken together, these findings support the view that the interplay of several co-inhibitory mechanisms rather than the PD-L1/PD-1 axis alone lead to PDAC immune evasion. Accordingly, targeting only one co-inhibitory axis by antibody blocking is not effective in improving tumor elimination and thereby the clinical situation of PDAC patients, rather than combinational targeting approaches of different co-inhibitory receptors or ligands.

Our stroma-enriched 3D co-culture systems well reflect several conditions in PDAC and its liver metastases regarding PD-L1 expression in PDAC and stromal cells as well as TME composition regarding macrophages and the effector phenotype of CD8+ T cells. However, it cannot fully mimic the complete spatial composition and dynamic changes in PDAC TME, e.g. several other determinants of immune evasion were not considered, e.g., the presence of ECM or additional immunosuppressive stromal cells such as myofibroblasts. Furthermore, it has to be critically mentioned that we cannot provide any reliable information on whether and to which extent CD8+ T cells and macrophages infiltrate into PDAC spheroids and whether ICI treatment impacts on it. For obtaining clear information regarding the spatial distribution of immune and PDAC cells in our spheroids, studies are planned to generate FFPE sections from spheroids for immunohistochemical stainings as performed with patient derived tissues. Additionally, T cells and macrophages were isolated and generated from PBMCs of healthy donors, thus being from another donor as the used PDAC cell lines. Accordingly, well defined time periods of co-cultures of CD8+ T cells and mono- and co-culture spheroids had to be used in order to avoid allogeneic T cell reactions. Furthermore, PBMCs of healthy donors might exhibit a different composition and activation status compared to PBMCs of PDAC patients (60). To overcome these limitations and to validate the findings obtained by this model system, future studies are planned to use PDAC cells and tumor associated macrophages from PDAC tissues as well as and PBMCs/CD8+ T cells from the same PDAC patient. However, here the challenge will be to obtain sufficient cell numbers of all cell populations analyzed. Furthermore, it would be interesting to analyze whether and how ICI treatment impacts polarization and effector phenotype of macrophages as well as plasticity of PDAC cells in order to get a comprehensive insight into ICI mediated effects on the entire TME. Finally, the results of the IHC stainings of matching primary tumors and liver metastases provided already valuable and unique insight into PD-L1 expression and the tumor stroma interplay during malignant progression of PDAC. Of note, metastases are not routinely resected and especially matched samples of primary tumors and metastases are very rare, so that this small collective has be considered very valuable but a validation of these findings with more matching tissue samples is certainly needed. Despite these limitations, our study revealed novel insights into the interplay of PDAC cells and macrophages in the presence of ICI treatment.

## Conclusion

Our study revealed that PD-L1 expression in primary PDAC correlates with PD-L1 expression in liver metastases with macrophages being a main source of PD-L1 expression. These cells can be predominantly found at the invasion fronts in primary tumors as well as in liver metastases presenting a barrier for CD8+ T cells which were also mainly detectable at the invasive fronts. This *in situ* condition was well mimicked by a 3D co-culture spheroid model using two PDAC cell lines with different PD-L1 expression status as well as different ratios and types of macrophages. In line with the *in situ* findings, macrophages were the main source of PD-L1 expression, however, the effector phenotype of CD8+ T cells was not impaired which was not associated with PDAC cell death induction. Despite the considerable PD-L1 and PD-1 expression in the different spheroid models, treatment with PD-1 and PD-L1 inhibitors Pembrolizumab and Durvalumab, respectively, as well as pre-treatment with Gemcitabine did neither boost the CD8+ T cell effector phenotype nor increased PDAC cell death. Despite its limitations, our study is in line with the view that the PD-1/PD-L1 axis may not be the main immunosuppressive mechanism of T cell mediated tumor immunity in PDAC.

## Data availability statement

The original contributions presented in the study are included in the article/**Supplementary Material**. Further inquiries can be directed to the corresponding author.

## Ethics statement

The studies involving human participants were reviewed and approved by Ethics committee of the Medical Faculty of Kiel University, Schwanenweg 20, 24105 Kiel, Germany and the ethics committee of the Friedrich-Alexander Universität Erlangen-Nürnberg, Krankenhausstr. 12 91054 Erlangen, Germany. The patients/participants provided their written informed consent to participate in this study.

## Author contributions

Conceptualization: TD, SB and SS; methodology: TD, SB, SR, SK and SH; resources: DW, CP, ME, AH, CR, HS, SS; writing—original draft preparation: TD, SB and SS; writing—review and editing: SR, SK, SH, HS, DW, CP, ME, AH, CR and AW; visualization: TD; funding acquisition: AMW, SS. All authors contributed to the article and approved the submitted version.
