# Supplementary figures and images for: Expression and role of the immune checkpoint regulator PD-L1 in the tumor-stroma interplay of pancreatic ductal adenocarcinoma
Source: Front Immunol. 2023 Jun 28;14:1157397. doi: 10.3389/fimmu.2023.1157397 (PMC10337136; doi:10.3389/fimmu.2023.1157397)

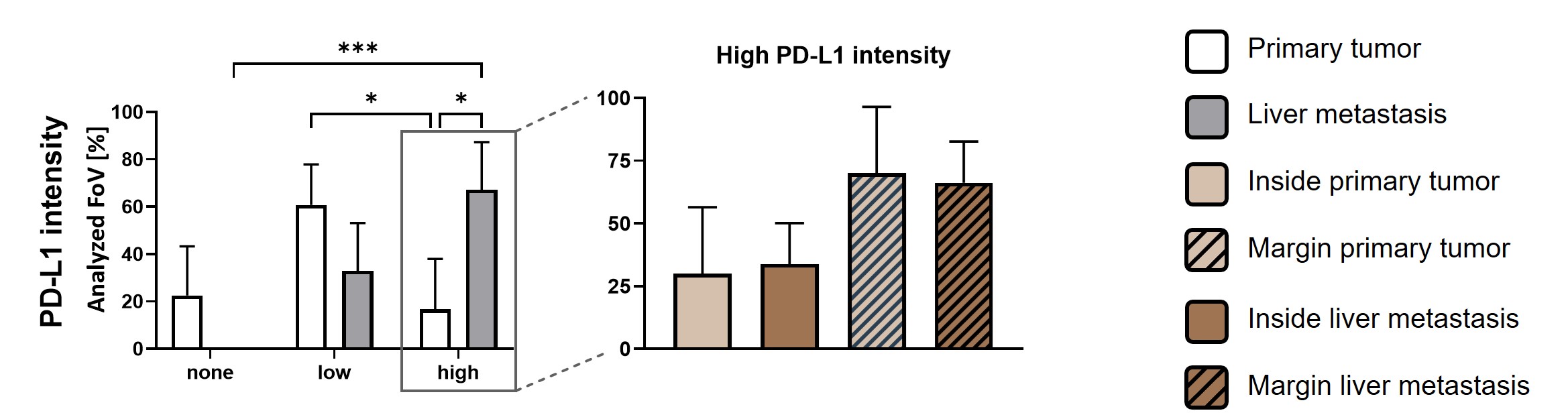

Supplement: Supplementary file 1 [file Image_1.jpeg]

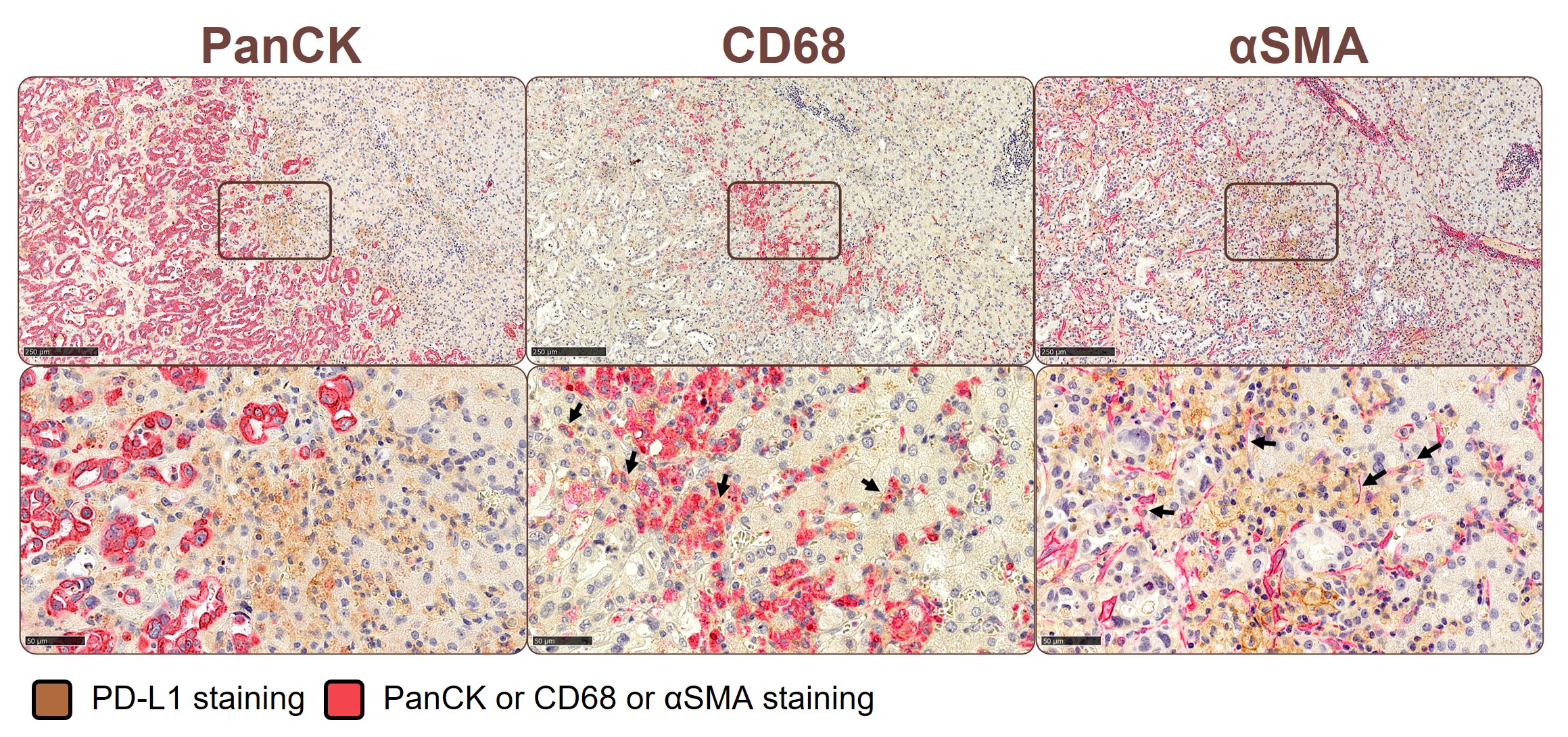

Supplement: Supplementary file 2 [file Image_2.jpeg]

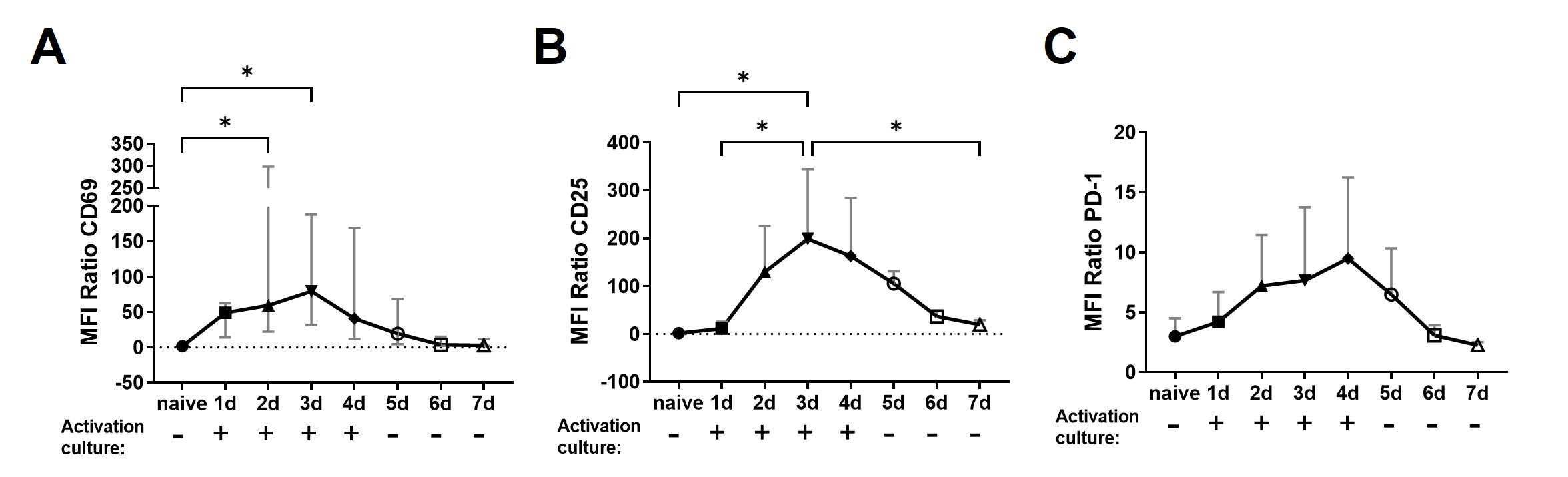

Supplement: Supplementary file 3 [file Image_3.jpeg]

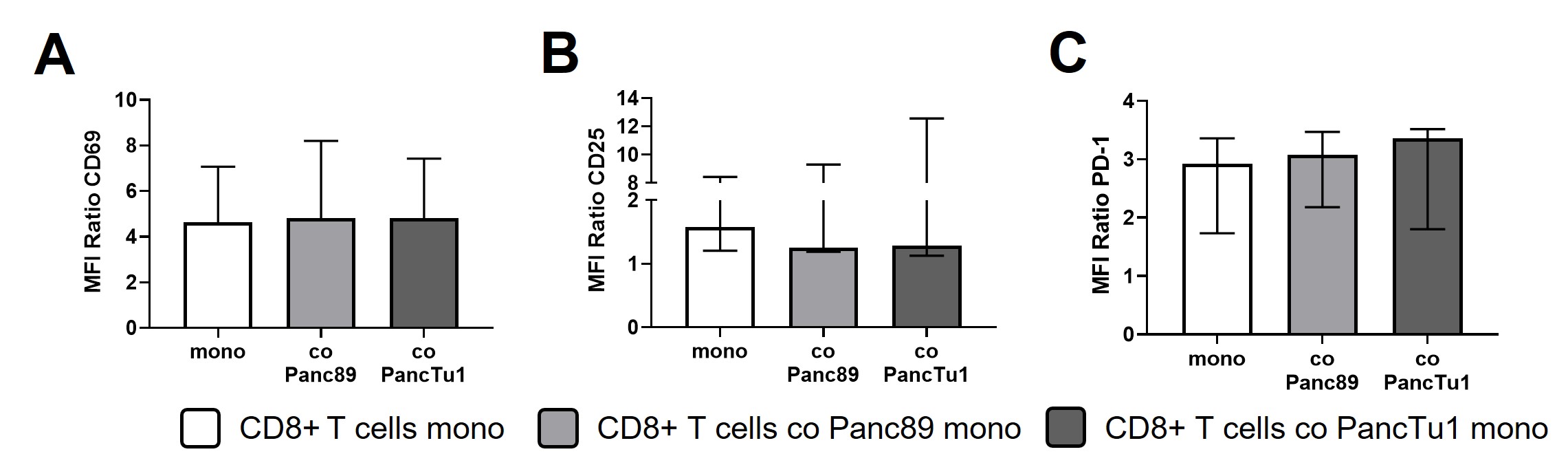

Supplement: Supplementary file 4 [file Image_4.jpeg]

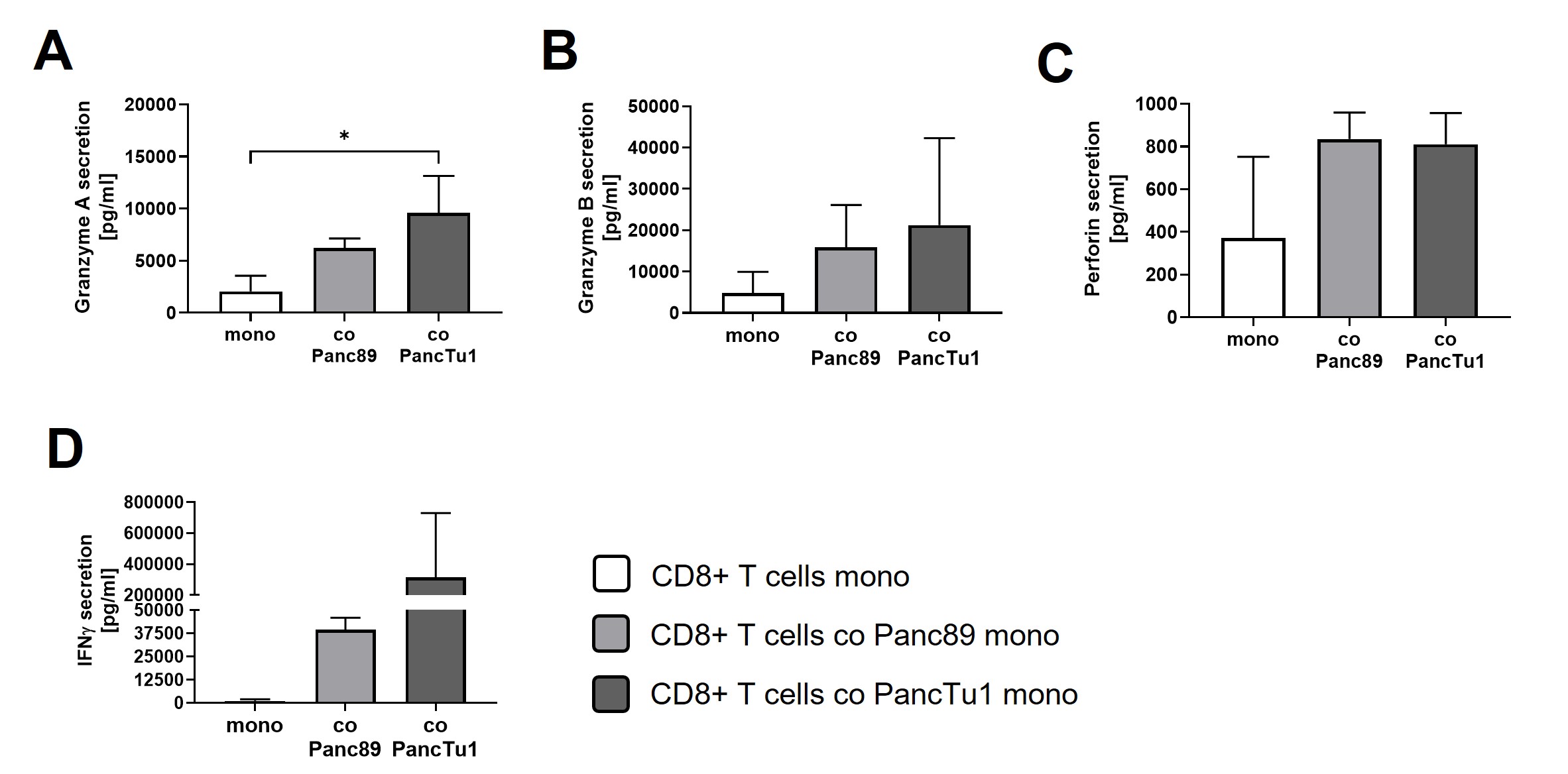

Supplement: Supplementary file 5 [file Image_5.jpeg]

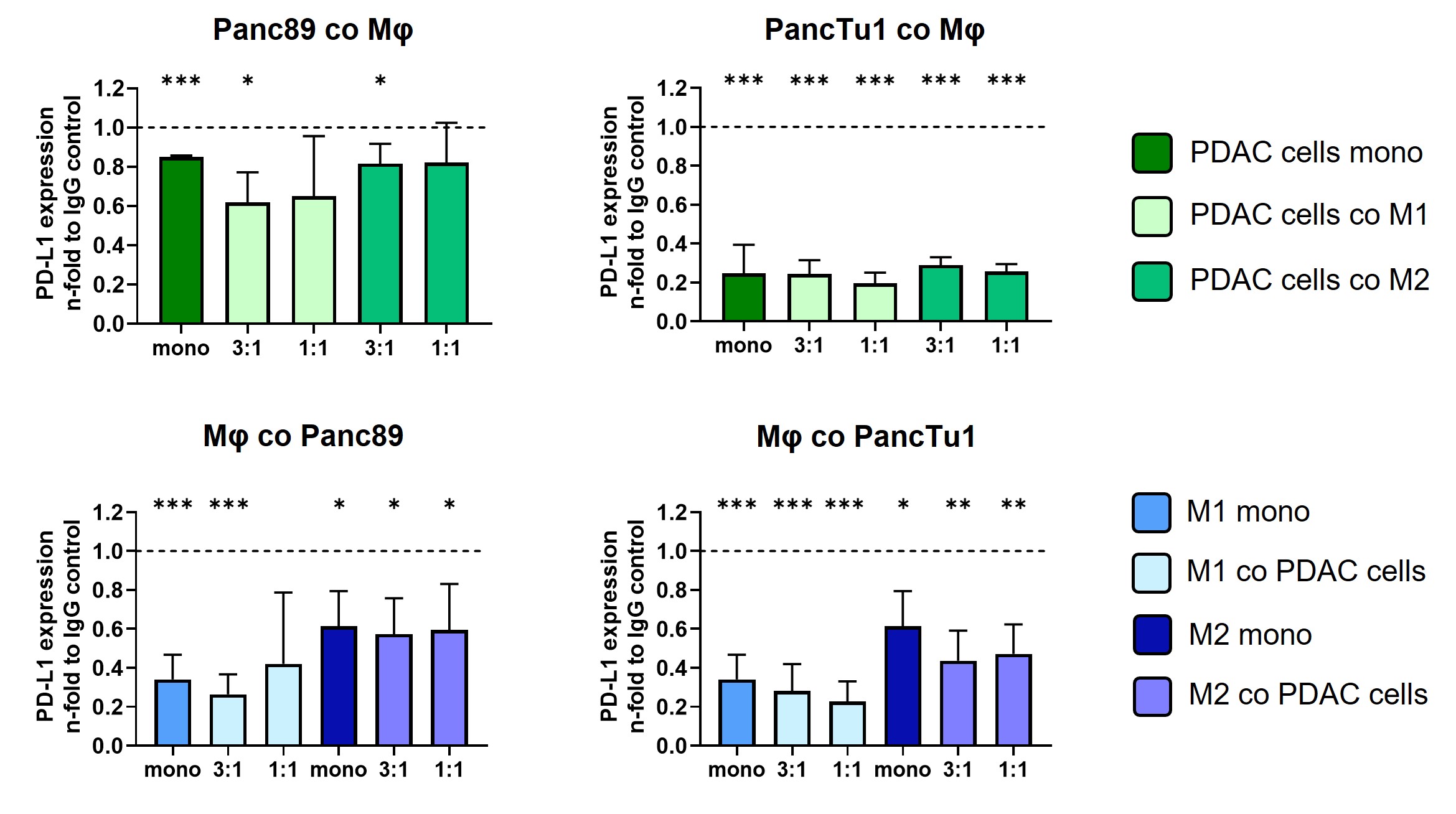

Supplement: Supplementary file 6 [file Image_6.jpeg]

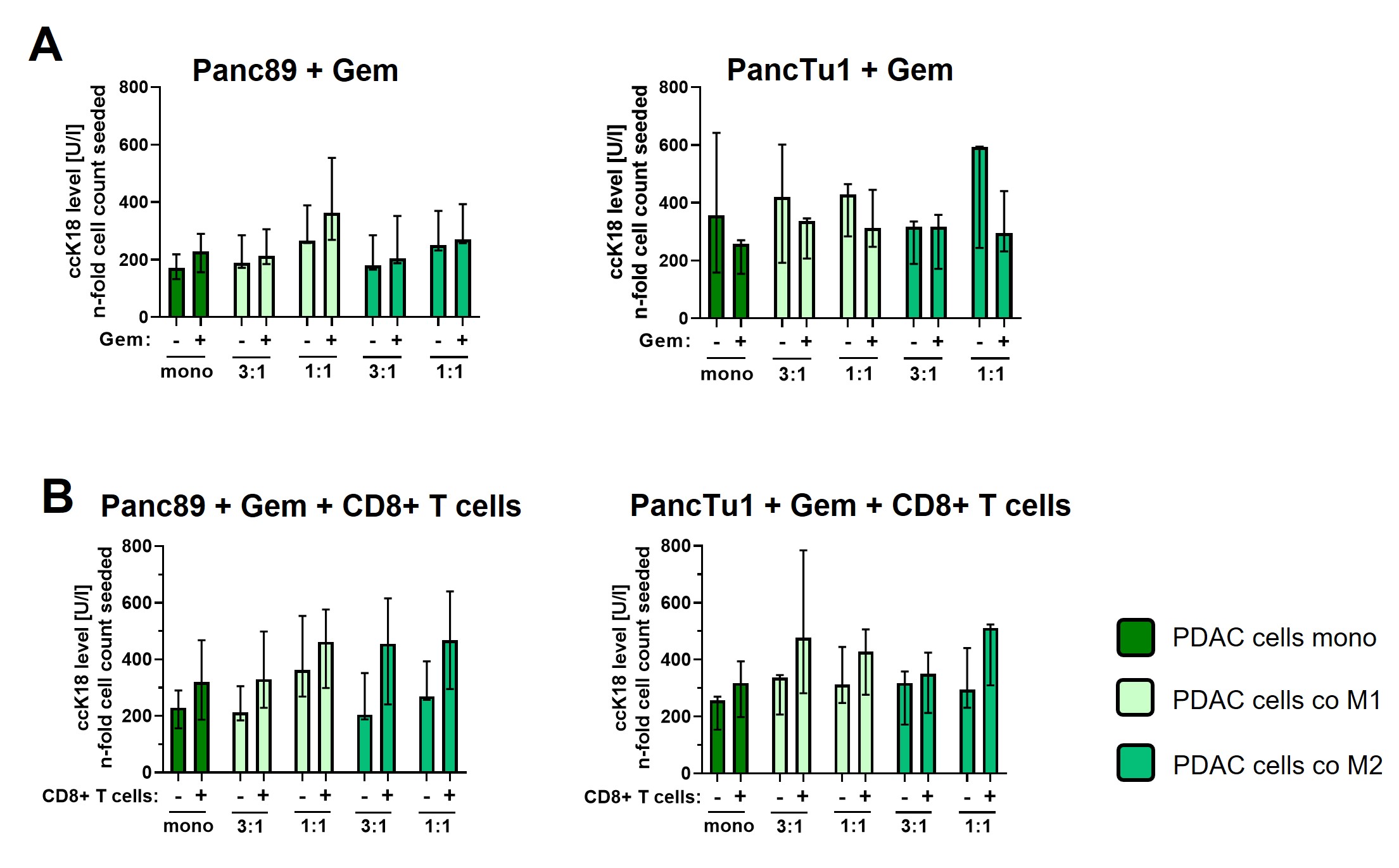

Supplement: Supplementary file 7 [file Image_7.jpeg]
